# The cathelicidin protein CRAMP is a potential atherosclerosis self-antigen in ApoE(-/-) mice

**DOI:** 10.1371/journal.pone.0187432

**Published:** 2017-11-01

**Authors:** Peter M. Mihailovic, Wai Man Lio, Juliana Yano, Xiaoning Zhao, Jianchang Zhou, Kuang-Yuh Chyu, Prediman K. Shah, Bojan Cercek, Paul C. Dimayuga

**Affiliations:** Oppenheimer Atherosclerosis Research Center, Division of Cardiology, Cedars-Sinai Heart Institute, Los Angeles, California, United States of America; Centro Cardiologico Monzino, ITALY

## Abstract

Auto-immunity is believed to contribute to inflammation in atherosclerosis. The antimicrobial peptide LL-37, a fragment of the cathelicidin protein precursor hCAP18, was previously identified as an autoantigen in psoriasis. Given the reported link between psoriasis and coronary artery disease, the biological relevance of the autoantigen to atherosclerosis was tested in vitro using a truncated (t) form of the mouse homolog of hCAP18, CRAMP, on splenocytes from athero-prone ApoE(-/-) mice. Stimulation with tCRAMP resulted in increased CD8+ T cells with Central Memory and Effector Memory phenotypes in ApoE(-/-) mice, differentially activated by feeding with normal chow or high fat diet. Immunization of ApoE(-/-) with different doses of the shortened peptide (Cramp) resulted in differential outcomes with a lower dose reducing atherosclerosis whereas a higher dose exacerbating the disease with increased neutrophil infiltration of the atherosclerotic plaques. Low dose Cramp immunization also resulted in increased splenic CD8+ T cell degranulation and reduced CD11b+CD11c+ conventional dendritic cells (cDCs), whereas high dose increased CD11b+CD11c+ cDCs. Our results identified CRAMP, the mouse homolog of hCAP-18, as a potential self-antigen involved in the immune response to atherosclerosis in the ApoE(-/-) mouse model.

## Introduction

Atherosclerosis is a chronic disease linked to auto-immune, pro-inflammatory processes potentially involving self-antigens [1]. Alterations of the host immune response involved in the disease process remains a growing field of study, and increasing evidence supports a role for self-reactive immune activation in atherosclerosis [2–5]. Control of self-reactivity by immune homeostasis is mediated in part by self-antigen processing and presentation through the MHC-I/CD8+ T cell pathway [6–8]. Under physiologic conditions, the host proceeds with this process without significant consequence. However, when stressed by pathologic inflammatory conditions, the host immune response is altered [9]. This process is thought to play a role in chronic diseases in humans [10,11]. Thus, the inflammatory response in coronary artery disease (CAD) may inflict stress upon the host leading to alterations in normal MHC-I/self-peptide immune responses.

Auto-immune, inflammatory diseases such as psoriasis have been linked to atherosclerotic cardiovascular diseases. After adjusting for key risk factors, early atherosclerosis was found to be highly prevalent in psoriasis patients compared to controls [12]. CAD patients have a two-fold higher prevalence of psoriasis [13]. LL-37, the cationic antimicrobial peptide fragment of the cathelicidin protein hCAP-18 [14], is an auto-immune T cell antigen in psoriasis patients [15]. The reported auto-immune response to LL-37 in psoriasis may be significant in CAD given the increased risk of myocardial infarction in psoriasis patients [16]. In support of these findings, LL-37 is found in atherosclerotic plaques [17,18], and prognosis after first-time myocardial infarction is significantly impaired in patients with psoriasis [19], suggesting that auto-immunity to LL-37 or other hCAP-18 fragments may be involved in atherosclerosis. In addition to its antimicrobial properties, LL-37 is a key component of neutrophil extracellular traps (NETs) [20] and has immune-modulatory functions [21].

To investigate the potential role of immune auto-reactivity to hCAP-18 in atherosclerosis, we designed experiments with the Apolipoprotein E(-/-) [ApoE(-/-)] mouse model of atherosclerosis using the mouse homolog of hCAP-18, called CRAMP (Cathelicidin Related Antimicrobial Peptide). The role of this potential auto-reactive antigen in atherosclerosis was tested using immunization as a strategy to manipulate the auto-immune response in ApoE(-/-) mice.

## Materials and methods

### Generation of truncated peptide for in vitro stimulation

The CRAMP protein was analyzed using an epitope prediction site to determine possible epitopes for antigenicity (www.syfpeithi.de; S1 Fig). Regions with low predicted binding scores to the mouse H2-K^b^ and H2-D^b^ alleles, which are the 2 alleles found in the C57Bl6 strain background of the ApoE(-/-), were excluded and a truncated(t) version of the peptide (tCRAMP) was synthesized (LifeTein) in order to test regions of CRAMP that were predicted to have high CD8+ T cell immune reactivity (S1 Fig).

### Animal protocol

The experimental protocols were approved by the Institutional Animal Care and Use Committee of Cedars-Sinai Medical Center. All mice were housed in a fully accredited animal facility, kept on a 12-h day/night cycle, and had unrestricted access to water and food. All experiments in the study were performed using male mice. Male ApoE(-/-) mice were purchased from Jackson Laboratories (Bar Harbor, ME). ApoE(-/-) mice were crossed with FoxP3^GFP^ mice on the C57Bl6 background (generously provided by Dr. Talal Chatila, UCLA). Male ApoE(-/-)FoxP3^GFP^ were used for regulatory T cell and mechanistic studies. Euthanasia was performed by overdose of inhalation anesthesia until death and assured by bilateral pneumothorax followed by tissue collection.

### In vitro stimulation of splenocytes with tCRAMP

The truncated peptide was used to stimulate splenocytes from 13 week old ApoE(-/-) mice fed with either normal chow (NC) or high fat diet (HC) consisting of 0.15% cholesterol, 21% fat (TD.88137, Envigo). The splenocytes from ApoE(-/-) mice were stimulated in a 37°C / 5% CO2 incubator with tCRAMP for 24 or 48 hours at a dose of 2 or 20μg/mL in RPMI medium (Thermo Fisher) supplemented with 10% Heat Inactivated FBS (Omega Scientific), antibiotic-antimycotic (Gibco) and β-mercaptoethanol (Sigma). Mouse Serum Albumin peptide (Abcam) was used as control. Albumin is an abundant serum protein with the peptide having only moderate binding prediction scores for H2-D^b^ and H2-K^b^ (S2 Fig).

For cell proliferation, splenocytes were stained with CFSE (2.5μM) and stimulated with tCRAMP (20μg/ml) for 4 days. Cells were harvested and stained with fluorescent CD4 or CD8b antibodies and analyzed using a BD LSR Fortessa apparatus.

### Plaque T cell stimulation with tCRAMP

ApoE(-/-) mice at 19 weeks of age were fed HC for 6 weeks until 25 weeks of age and the aortas were collected at euthanasia for enzymatic digestion. Mice were used at this age in order to assure presence of atherosclerotic plaques in the aorta. Aorta pooled from 6 mice were incubated for 20 minutes in 37°C sterile RPMI 1640 medium with 0.25 mg/ml Collagenase (Sigma-Aldrich), 0.125 mg/ml Elastase (Sigma-Aldrich), and 60 U/ml Hyaluronidase. After enzymatic digestion, the cells were strained through a 40 μm Nylon cell strainer (Falcon) to obtain single cell suspension. The cells were cultured in triplicates for 24h in in a 37°C / 5% CO2 incubator with tCRAMP at a dose of 20μg/mL in RPMI medium.

### Flow cytometry

Harvested cells were washed in PBS and stained for viability (LIVE/DEAD Fixable Violet stain) and surface markers listed in Table 1. For intracellular cytokine staining, Brefeldin A (eBioscience) was added to the cultured cells 4 hours before the staining procedure. After cell surface staining, cell membranes were permeabilized with Fixation/Permeabilization buffer (eBioscience) and subjected to intracellular staining using standard protocol with antibodies in Table 1. For cells collected from enzymatic digestion of the aorta, CD3 antibody was used to mark T cells. Flow cytometry was performed using a BD LSR Fortessa apparatus. General gating for analysis excluded cell doublets and non-viable cells. Specific gating strategies are described in the results section.

**Table 1:** Antibodies used for flow cytometric analysis.

Antibodies	Clone
anti-CD3e (BD Horizon)	145-2C11
anti-CD4 (BD Horizon)	GK1.5
anti-CD8a (eBioscience)	53–6.7
anti-CD8b (eBioscience)	H35-17.2
anti-CD11b (eBioscience)	M1/70
anti-CD11c (eBioscience)	N418
anti-CD25 (eBioscience)	PC61.5
anti-CD44 (eBioscience)	IM7
anti-CD49b (eBioscience)	DX5
anti-CD62L (eBioscience)	MEL-14
anti-CD107a (Biolegend)	1D4B
anti-CD122 (eBioscience)	5H4
anti-CD317/PDCA-1 (eBioscience)	129c
anti-IFN-γ (eBioscience)	XMG1.2
anti-IL-12 (eBioscience)	C17.8
anti-TNF-α (Biolegend)	MP6-XT22

### Cramp peptide synthesis for immunization

The peptide sequence excluding the first 37 amino-acids of the protein (final length is 136 aa) was synthesized (LifeTein) and used for immunization studies, referred to as Cramp throughout the report (S1 Fig). The first 37 amino-acids were excluded due to the low MHC-I binding prediction scores of that region.

### Immunization of ApoE(-/-) mice with Cramp peptide

The peptide was formulated with Adjuphos (Brenntag; 12.5 μl of a 2% solution) and 10μg MPLA (MPLA-SM VacciGrade, InvivoGen) as adjuvant mixture, in a final volume of 200μl with PBS as vehicle. Controls were PBS alone or adjuvant alone (Adjuphos and MPLA with PBS at matched volumes). Male mice were immunized subcutaneously at a dose of 20μg or 100μg of the peptide at 7, 10, and 12 weeks of age. Two different doses were used to address the potential role of antigen dose in self-reactive immune responses [9,22–24]. At 13 weeks of age, mice were fed high fat diet until euthanasia at 25 weeks of age. Mechanism of action studies were performed utilizing ApoE(-/-)FoxP3^GFP^ mice.

### Tissue harvesting and preparation

At euthanasia the mouse hearts were harvested and embedded in OCT compound (Tissue-Tek) for cryo-sectioning. Whole aortas were cleaned, fixed in Histochoice (Amresco) and stained with Oil-red-o to assess the extent of atherosclerosis en face as described previously [25,26].

### Immunohistochemical and lipid staining of aortic sinus plaques

The aortic sinus sections were stained with Oil-red-o, MOMA-2 antibody (BioRad), anti- CD3 and Ly6G (Biolegend) antibodies to identify lipids, macrophages, T cells, and neutrophils, respectively using standard immunohistochemical protocol. Three slides per animal in 5 slide intervals were used for each staining parameter. Results from the 3 slides were averaged per animal for the respective parameter. Secondary antibody conjugated to HRP with AEC as color substrate was used for detection and sections were counter-stained with hematoxylin. Omission of primary antibody served as negative control. Computer assisted morphometric analysis by ImagePro software (Media Cybernetics) was performed on samples as described previously [25,26]. The person performing the computer-assisted analysis was blinded to mouse treatment groups.

### Serum analysis

Anti-Cramp IgM and IgG titers were assessed using ELISA. Cramp peptide was coated onto 96- well plates at a concentration of 20μg/ml overnight. Wells were blocked with 1% BSA and diluted sera added into the coated wells (IgM 1:200 dilution; IgG 1:50 dilution) for 1 hour. Standard ELISA procedure was followed using HRP-conjugated goat anti-mouse IgM (Southern Biotech) or IgG (Thermo Scientific) and ABTS (Southern Biotech) as colorimetric substrate. Results are expressed as optical density (OD) absorbance units at 405 nm. Cholesterol levels were measured with a commercially available kit (Wako).

### T cell profile in immunized mice

Spleens from immunized and control mice were harvested one week after last booster injection (at 13 weeks of age) and the splenocytes were cultured with tCRAMP stimulation (20μg/ml) for various times as indicated in the figure legends. Cells were harvested and subjected to staining for flow cytometry as indicated above.

### CD8+ T cell degranulation

Spleens from immunized and control mice were harvested one week after last booster injection and splenocytes from experimental groups were cultured as described above. Fluorescent-conjugated antibody to granular membrane protein CD107a (Biolegend) was added to the cell culture prior to stimulation with peptide. The cultures were then incubated for 1 h at 37°C in 5% CO2, followed by incubation with transport inhibitor Monensin (eBioscience) for 4 hours. The cells were then harvested, washed with PBS and stained as described above.

### Statistics

All data are reported as mean ± SD. Based on normality test, data were analyzed using ANOVA followed by Newman-Keuls test for group comparison or Kruskal-Wallis test followed by Dunn’s multiple comparison test, respectively. Comparison between two groups was performed using T-test. A P value < 0.05 was considered significant.

## Results

### Dose-response and kinetics of T cell response to tCRAMP

The potential immunogenic role of CRAMP in atherosclerosis in the context of MHC-I/self-peptide T cell reactivity was tested first in vitro by 24h or 48h stimulation of splenocytes harvested from 13 week old naïve ApoE(-/-) mice fed normal (NC) using tCRAMP. Regions of low binding prediction for H2-D^b^ and H2-K^b^ were excluded from the synthesized peptide in order to include only peptide fragments that are potentially MHC-I reactive (see Methods and S1 Fig). Gating scheme for analysis is shown on S3 Fig.

**Fig 1:**
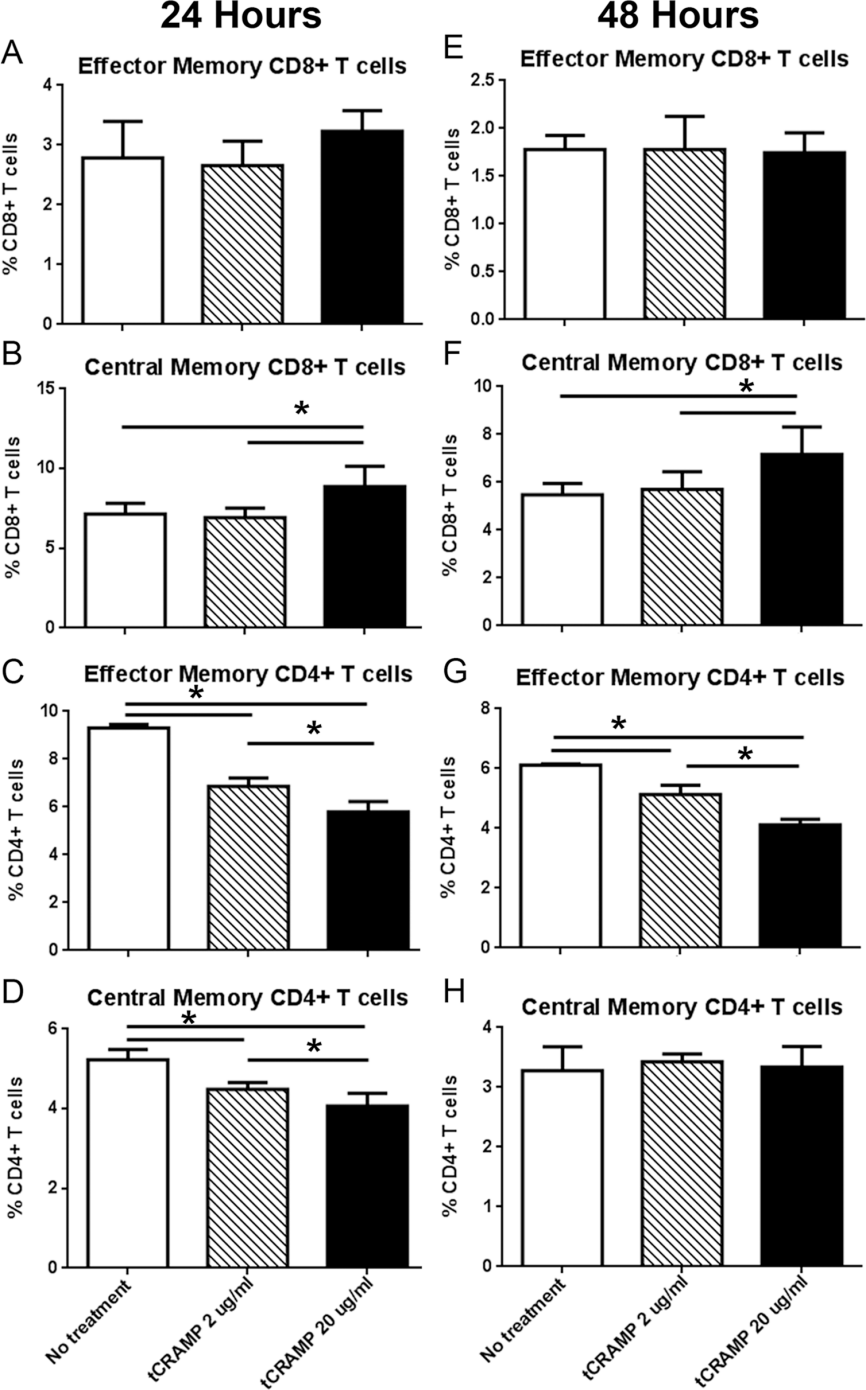
Response to tCRAMP stimulation of ApoE(-/-) splenocytes. Splenocytes from naïve 13 week old ApoE(-/-) mice were stimulated with 2 or 20μg/ml truncated(t) CRAMP peptide for 24 hours (A-D) or 48 hours (E-H). Analysis of cell stains was based on the gating scheme depicted in S3 Fig. Bars over graphed columns indicate statistical significance (P<0.05; N = 4 each).

**Fig 2:**
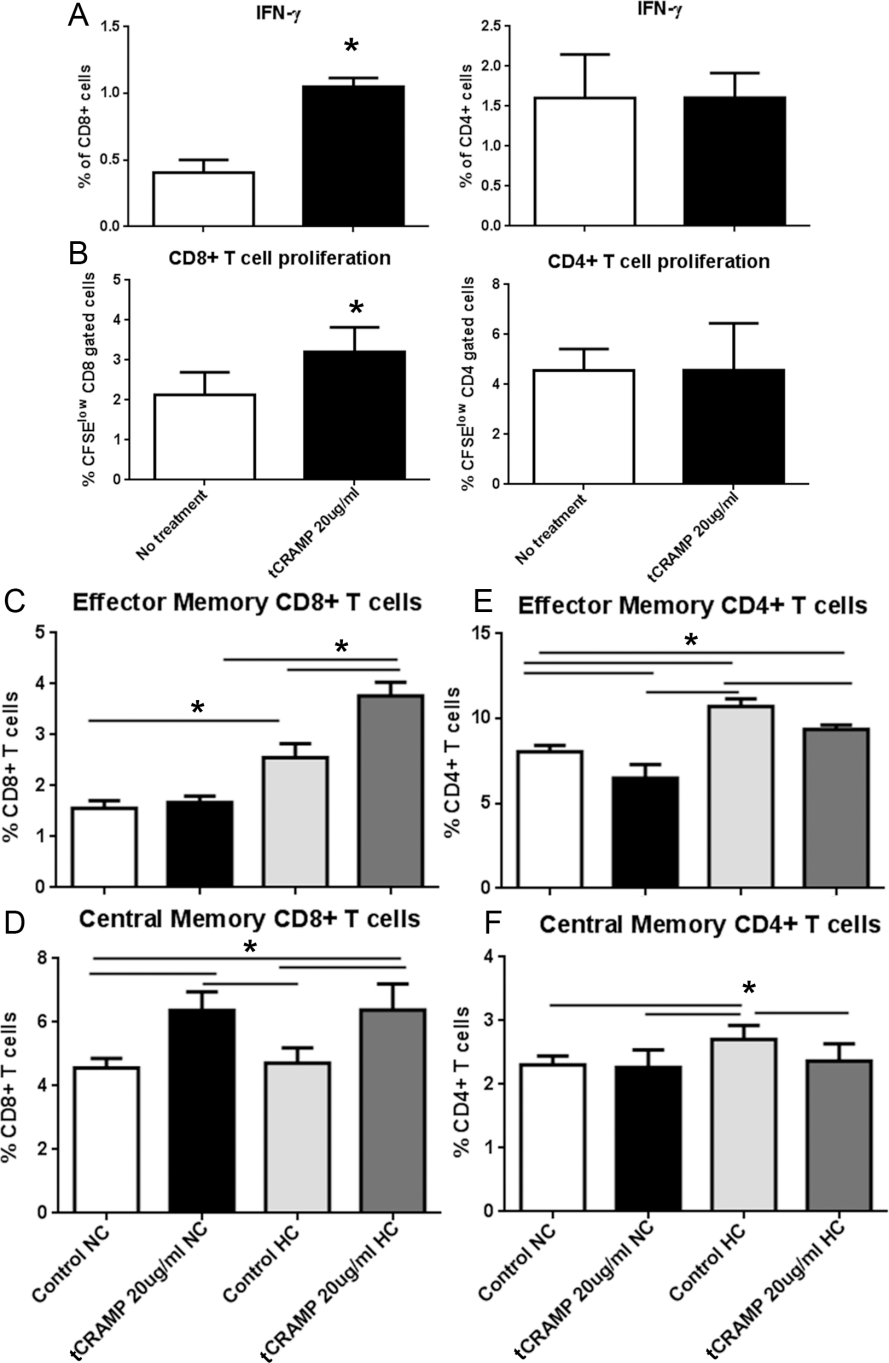
T cell response to tCRAMP and effect of high fat diet. Splenocytes from 13 week old naïve ApoE(-/-) mice were stimulated with 20μg/ml truncated(t) CRAMP peptide for 48 hours (A) or stained with CFSE and stimulated with CRAMP 4 days (B) then stained for intracellular IFN-γ or analyzed for cell proliferation, respectively. CD8+ T cells are on the left panels and CD4+ T cells are on the right panels. *P<0.05; N = 4 each. Splenocytes from mice fed normal chow (NC) or a high fat diet (HC) for 6 weeks starting at 7 weeks of age were treated with 20μg/ml tCRAMP for 48 hours (C-F). Analysis of cell stains was based on the gating scheme depicted in S3 Fig. Bars over graph columns indicate statistical significance (P<0.05; N = 4 each).

Stimulation with tCRAMP (20ug/ml) showed no effect on CD8+ T cells with Effector Memory (EM) markers (Fig 1A) but a significant increase in CD8+ T cells with Central Memory (CM) markers (Fig 1B) and a decrease in both EM and CM CD4+ T cells after 24 hours (Fig 1C and 1D). The effects persisted after 48 hours (Fig 1F and 1G). Cytokine expression and cell proliferation were also assessed after tCRAMP stimulation. There was increased CD8+IFN-γ+ T cells in splenocytes after 48 hours of tCRAMP stimulation, with increased CD8+ T cell proliferation 4 days after tCRAMP stimulation (Fig 2A and 2B, respectively). These results show reactivity to tCRAMP stimulation by CD8+ T cells that manifest memory cell markers.

Next we tested whether the immunologic reactivity would be altered by high fat diet (HC) feeding of naïve ApoE(-/-) mice for 6 weeks since pro-inflammatory HC feeding accelerates atherosclerotic lesion formation in this mouse model. HC mice had increased EM CD8+ T cells with 48 hour tCRAMP stimulation (Fig 2C). Similar to normal chow (NC) fed mice, tCRAMP stimulation of splenocytes from HC ApoE(-/-) mice showed a significant increase in CM CD8+ T cells (Fig 2D). The trend of reduced EM and CM CD4+ T cells was observed in NC as well as HC mice (Fig 2E and 2F). To determine if another self-protein derived peptide (S2 Fig) would provoke immune responses, a peptide fragment of mouse Albumin protein was used as a potential self-reactive antigen. Stimulation of splenocytes from ApoE(-/-) mice fed HC with mouse Albumin peptide had no effect (S4 Fig).

### Increased inflammation by tCRAMP stimulation in CD11b+ DCs in high fat diet fed mice

**Fig 3:**
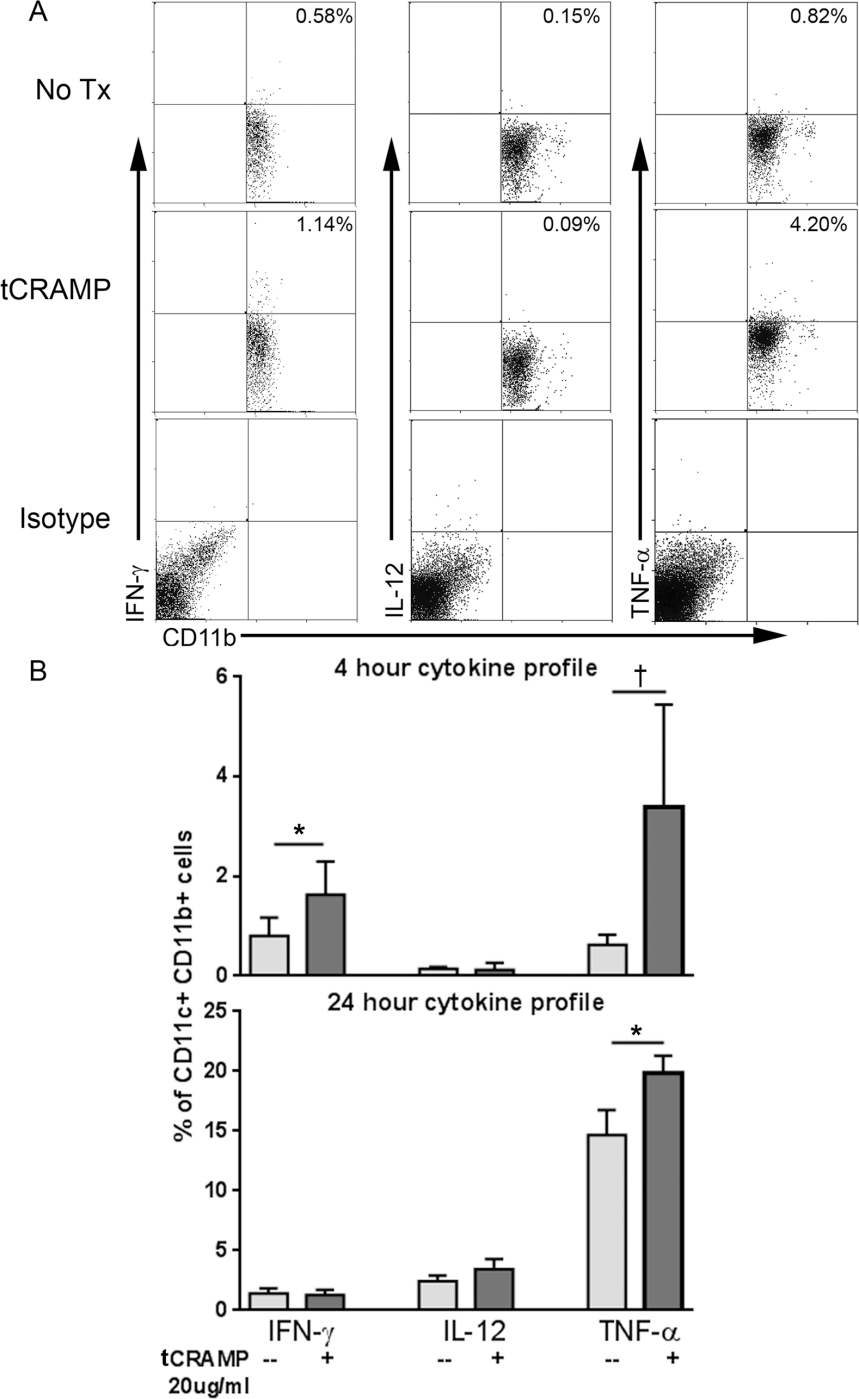
Inflammatory cytokine profile of CD11c+CD11b+ cDCs in mice fed high fat diet. Splenocytes from naïve mice fed a high fat diet were treated with 20μg/ml tCRAMP for 4 or 24 hours and stained for intracellular cytokines. Isotype staining was used as control. Gating scheme for CD11c+ DCs as depicted in S5 Fig. Representative scatter plots are shown (A). Results were plotted on bar graphs (B). *P<0.05; †P = 0.08. Splenocytes pooled from 2 mice and assayed in triplicates.

CD11c+CD11b+ cDCs are the dominant DC population in both human [27] and mouse plaques [28] and associated with worsening of the disease. However, the scant number of CD11c+ DCs in plaques was a technical limitation for subtype analysis of intra-cellular cytokine staining for flow cytometry. We therefore assessed the effect of tCRAMP stimulation on the CD11c+CD11b+ cDC population in splenocytes from naïve apoE-/- mice fed HC. Stimulation with tCRAMP resulted in increased pro-inflammatory profile of CD11c+CD11b+ cDCs which was evidenced by increased IFN-γ and TNF-α within 4 hours of stimulation, and persisted for 24 hours (Fig 3A and 3B). Gating scheme for CD11c+ DCs is shown in S5 Fig.

### tCRAMP reactive T cells in the aortic plaques

**Fig 4:**
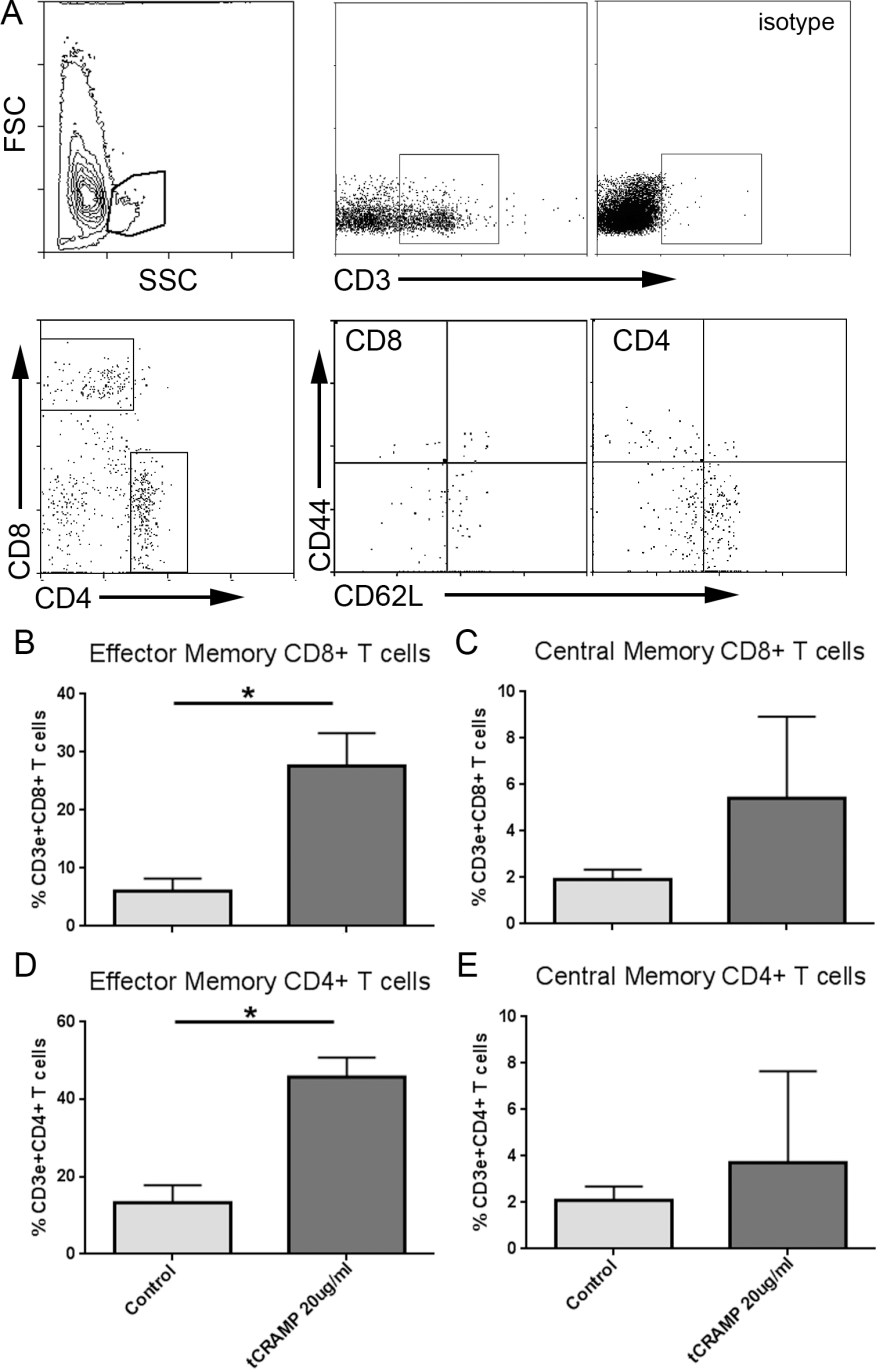
Plaque T cells are reactive to tCRAMP simulation. Aortas from ApoE(-/-) mice at 25 weeks of age were subjected to enzymatic digestion and stimulated for 24 hours with 20μg/ml tCRAMP. Size gate is shown after inclusion of singlets and exclusion of non-viable cells, followed by gating for CD3+ T cells (A). Isotype was used as staining control. Cells were plotted on CD4 vs CD8 and selected for subtype analysis. Results were plotted on bar graphs (B-E). Aortas from 6 mice were pooled and assayed in triplicates. Bars over graph columns indicate statistical significance (P<0.05).

To determine if CRAMP-reactive T cells were present in atherosclerotic plaques, aortas from 25-week old ApoE(-/-) mice were subjected to enzymatic digestion and the cells stimulated with tCRAMP for 24 hours. Stimulation with tCRAMP elicited a significant response in CD8+ Effector Memory (Fig 4A and 4B) and trending higher CD8+ Central Memory T cell population (Fig 4C). The self-peptide response to tCRAMP was observed in the CD4+ EM T cell population (Fig 4D) but with no significant change in CD4+ CM T cell population (Fig 4E). Thus, our results show reactivity to tCRAMP by CD8+ T cells that express memory markers, which is exacerbated by high fat diet in ApoE(-/-) mice.

### Differential effects of Cramp immunization dose on atherosclerosis in ApoE(-/-) mice

**Fig 5:**
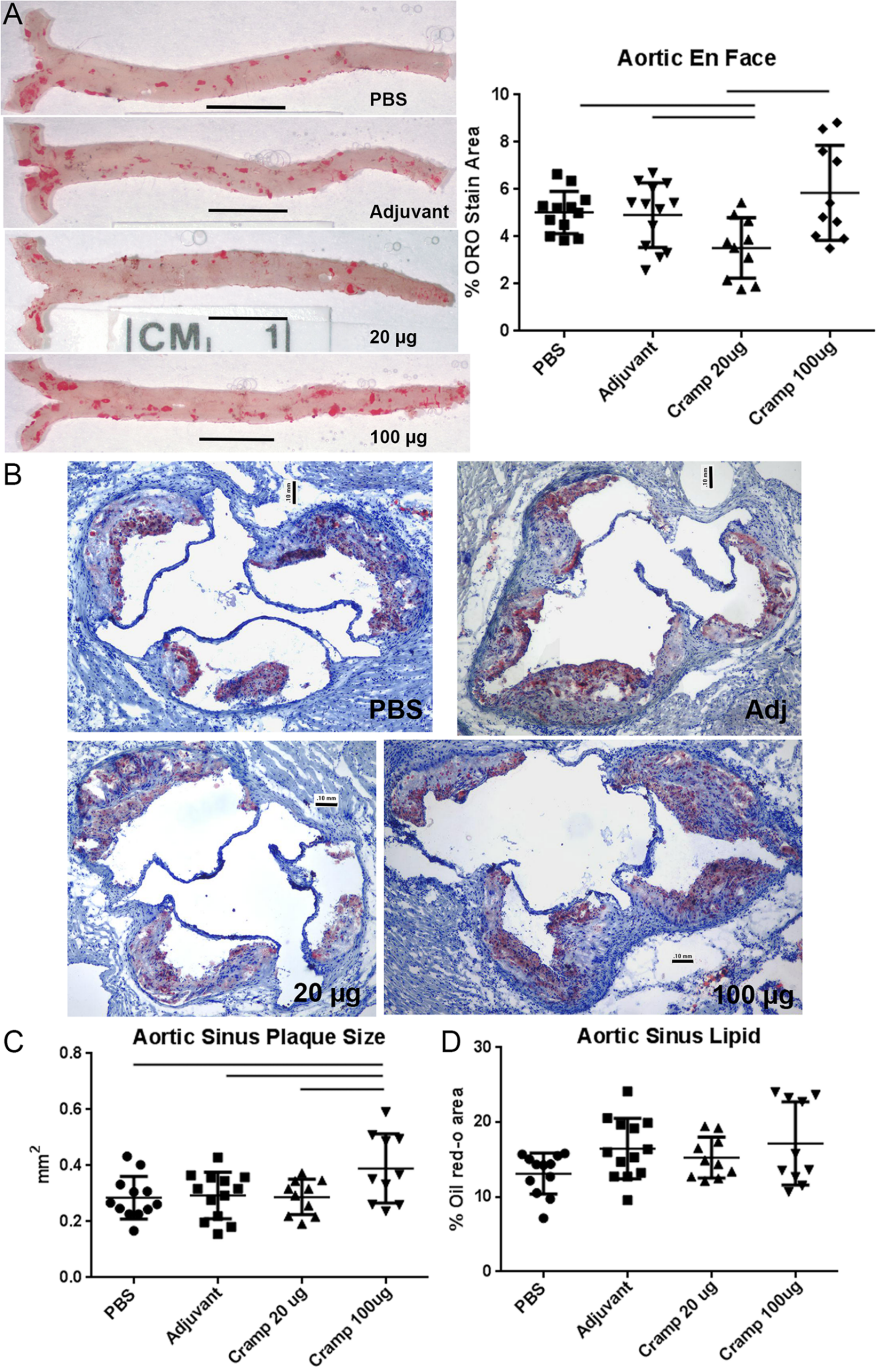
Differential effect of Cramp immunization on atherosclerosis. The peptide sequence excluding the first 37 amino-acids of the protein (final length is 136 aa, S1 Fig) was synthesized and used for immunization studies. ApoE(-/-) mice fed normal chow were immunized with either 20μg or 100μg of Cramp mixed with adjuvant at 7, 10, and 12 weeks of age then switched to high fat diet at 13 weeks of age. Representative photographs of aortas collected at euthanasia and subjected to en face oil red-o staining are shown for each experimental group (A, left panel), as labeled. Bars = 0.5 cm. Percent aortic plaque area of the aorta obtained by image analysis is plotted for each animal in the respective groups (A, right panel). Bars over graph columns indicate statistical significance; P<0.05. PBS N = 12; Adj N = 13; Cramp 20μg N = 10; Cramp 100μg N = 10. Plaque size and lipid presence were assessed in the aortic sinus of mice using oil red-o staining. Representative photographs are shown for each group as labeled (B). Bar = 0.1mm. Image analysis measurement of plaque size (C) and percent lipid area (D) were plotted. Bar over graph columns indicates statistical significance; P<0.05.

**Fig 6:**
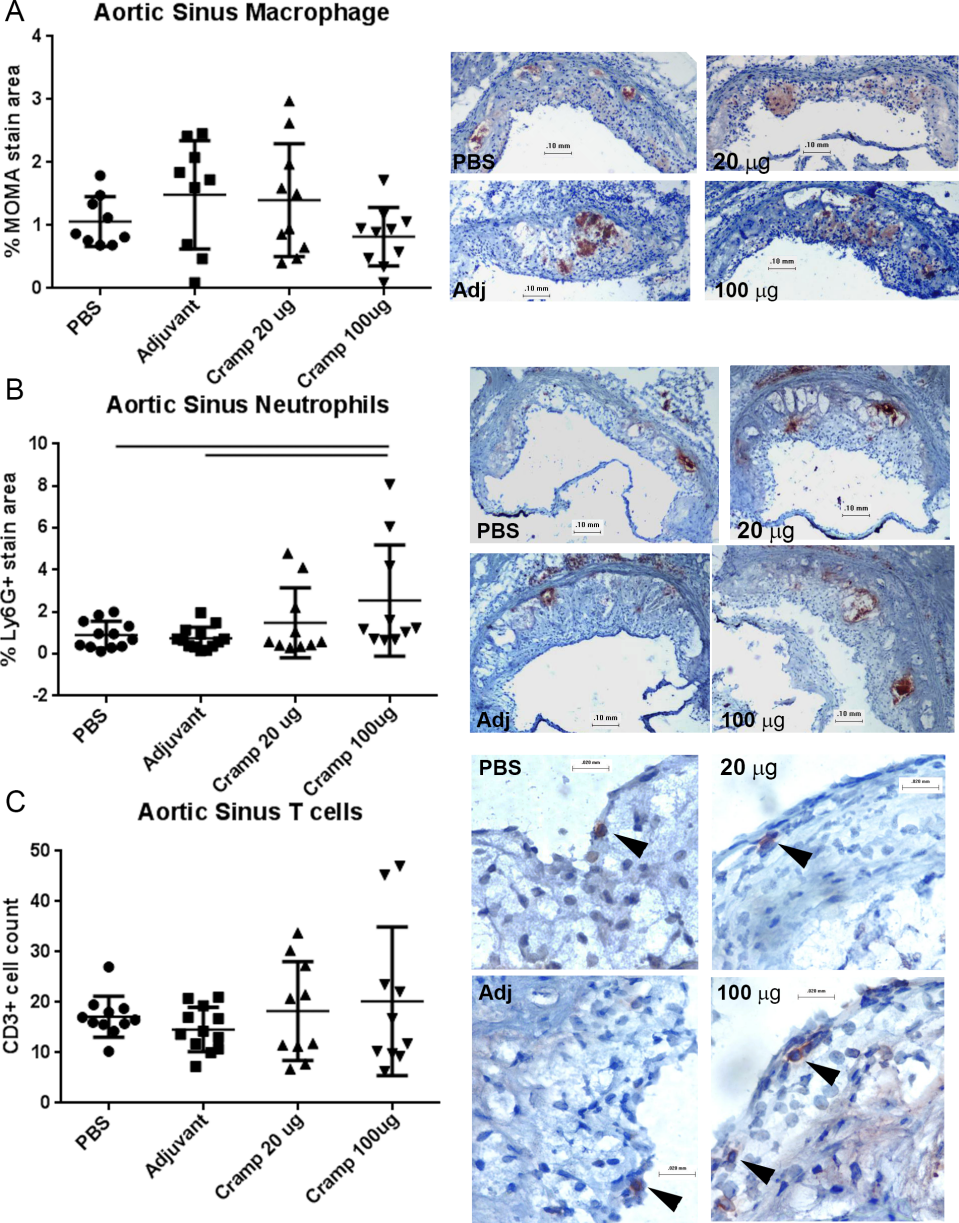
Effect of Cramp immunization on aortic sinus plaque inflammation. Aortic sinus plaque macrophage (A), neutrophil (B), and T cell (C) infiltration were assessed using immuno-histochemical staining. Representative photographs on the right are shown for each group as labeled. Top and middle photographs bar = 0.1mm; bar for bottom photograph = 0.02mm. Image analysis measurement of percent plaque stain area and cell count were plotted. Bar over graph columns indicates statistical significance; P<0.05. Negative staining controls are found in S6 Fig.

Given that tCRAMP provoked memory-like responses from reactive CD8+ T cells, we reasoned that the immunogenic regions are contained within or near the sequences predicted to have high MHC-I binding. tCRAMP was generated by editing out non-MHC-I binding regions and linking the remaining segments for the tCRAMP peptide. It is potentially foreign to the mice and therefore cannot be considered a self-peptide for immunization. However, we wanted to immunize the mice to alter the immune reactivity to the self-protein, and not potentially foreign peptide sequences in tCRAMP. Since the first 37 amino-acids that had low MHC-I binding prediction scores were also excluded from tCRAMP, it was a reasonable to assume that immune-reactivity to a significant portion of the CRAMP protein would still be attained. Using the peptide sequence that excluded the first 37 amino-acids of the protein (S1 Fig), ApoE(-/-) mice were immunized with 20μg or 100μg of Cramp peptide at 7, 10, and 12 weeks of age using Adjuphos with MPLA as adjuvant. Adjuvant or PBS alone served as controls. The mice were then fed with HC at 13 weeks of age and euthanized at 25 weeks of age. Overall atherosclerotic burden was assessed using aortic en face oil red-o staining (Fig 5A, left panel). Aortic plaque area was significantly decreased in the 20μg immunized mice compared to controls. The aortic plaque area in the 100μg dosed group was trending higher and was significantly increased compared to the 20μg dose (Fig 5A, right panel). The 100μg immunization dose also significantly increased aortic sinus plaque size (Fig 5B and 5C). There was no observed change in aortic sinus lipid staining (Fig 5B and 5D) or macrophage staining (Fig 6A) among the groups. There was significantly increased neutrophil infiltration of plaques in the 100μg dosed-group compared to controls (Fig 6B), and no significant difference in CD3+ T cell count in plaques among the groups (Fig 6C). The differential effects of immunization with the 20μg or 100μg doses on atherosclerosis suggested that modifying antigenic exposure to a self-peptide relevant in atherosclerosis can modulate the immune response to the self-antigen and change atherosclerotic plaque development.

### Serum cholesterol, anti-Cramp antibody response, and CD4+CD25+FoxP3+ T_reg_ cells

**Fig 7:**
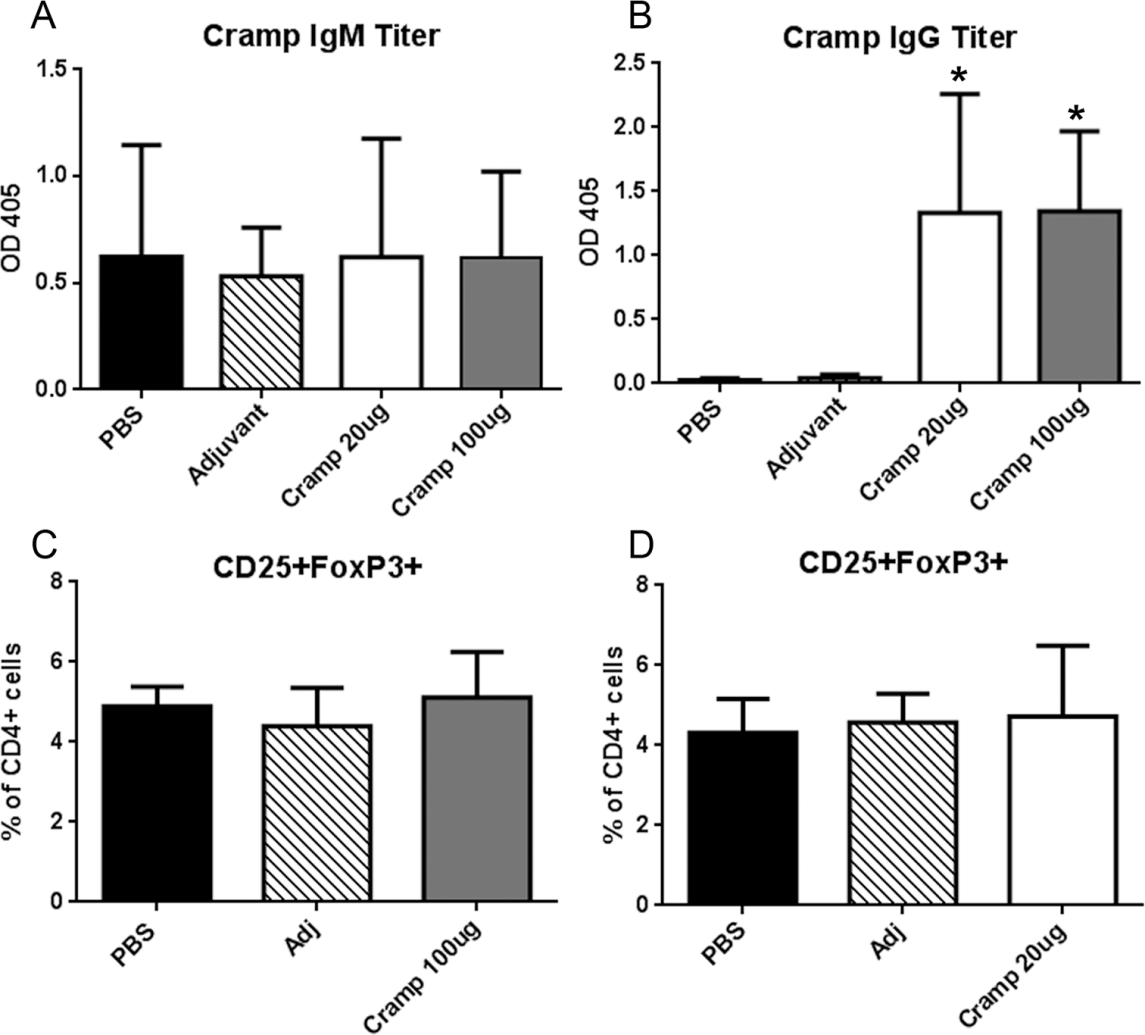
Anti-Cramp Ig titers and FoxP3+ T_reg_ cells in immunized mice. Serum anti-Cramp IgM titers (A) and anti-Cramp IgG titers (B) were measured and plotted. *P<0.05 vs PBS and Adjuvant. FoxP3+ T_reg_ cells were assessed in ApoE(-/-)FoxP3^GFP^ mice. Splenocytes from Cramp 100μg dosed mice (C) or Cramp 20μg dosed mice (D) were collected one week after the last booster injection and challenged with 20μg/ml tCRAMP for 24 hours and subjected to CD4 and CD25 staining and detection of FoxP3+ T_reg_ cells using GFP fluorescence shown in S3 Fig.

We examined the humoral response to Cramp immunization at the 25-week time point. Anti-Cramp IgM antibody presence was unchanged in all groups compared to the PBS control (Fig 7A). Increased anti-Cramp IgG (Fig 7B) occurred in both Cramp immunization doses to a similar degree suggesting that the differential effects on atherosclerosis are unlikely mediated by the antibody response. No differences were observed in serum cholesterol levels among the groups (PBS = 1533±389 mg/dL; Adjuvant = 1475±235 mg/dL; Cramp 20μg = 1508±384 mg/dL; Cramp 100μg = 1392±497 mg/dL).

Responses to self-antigens are governed by peripheral tolerance mechanisms mediated in part by FoxP3+ T regulatory (T_reg_) cells. To elucidate the mechanism of action and the dichotomy of responses from the 20μg and 100μg immunization doses, T_reg_ cell response was examined utilizing ApoE(-/-)FoxP3^GFP^ mice. ApoE(-/-)FoxP3^GFP^ mice were immunized using the schedule described in the Methods section and euthanized one week after 2^nd^ booster dose. No differences were observed in CD4+CD25+FoxP3+ T_reg_ cells among the groups (Fig 7C and 7D). Thus, the observed differential activation occurred despite similar CD4+CD25+FoxP3+ T_reg_ cell counts.

### Antigen dose determines T cell activation profile

**Fig 8:**
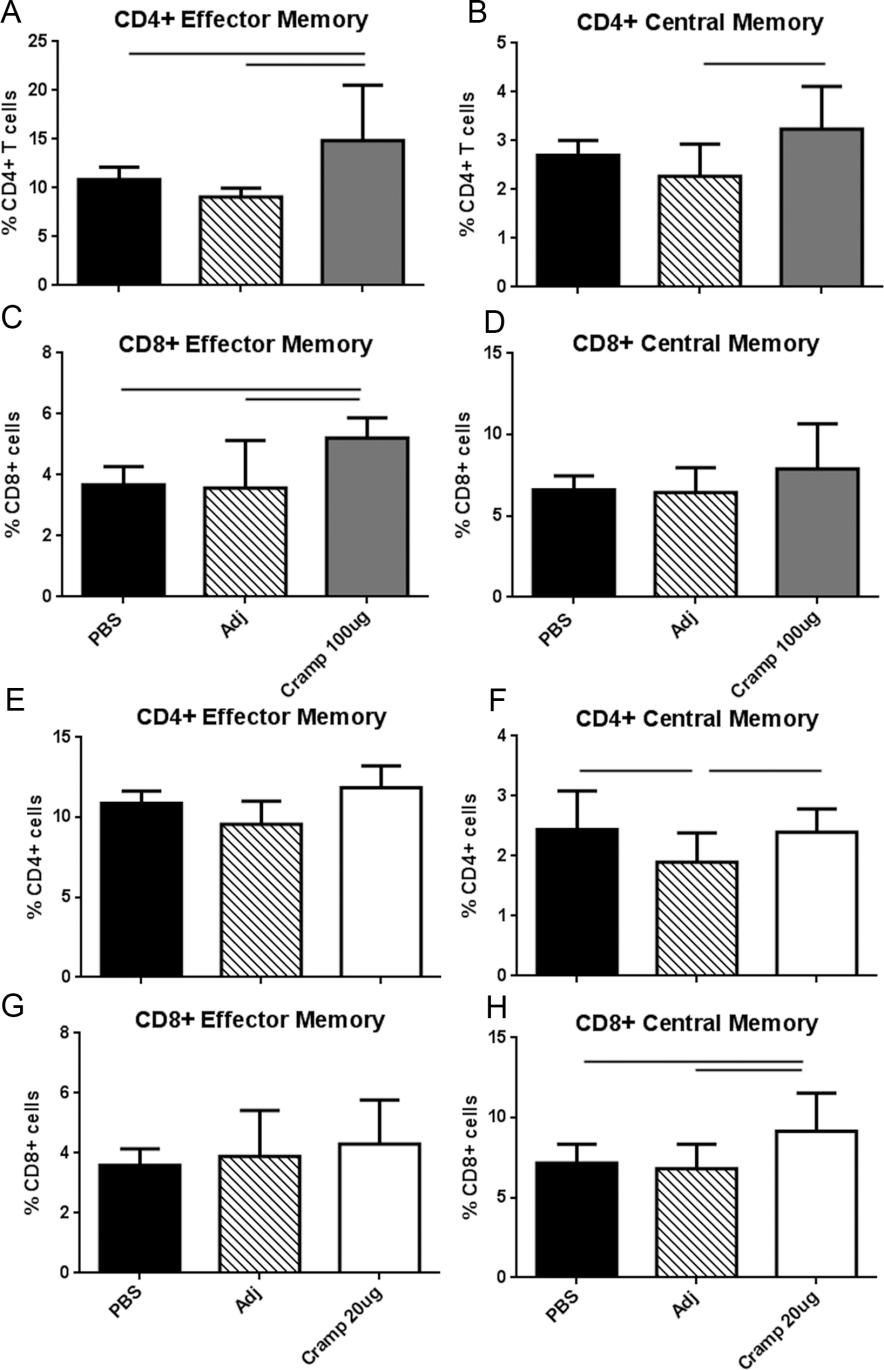
T cell activation in Cramp-immunized mice. Splenocytes from Cramp 100μg dosed mice (A-D) or Cramp 20μg dosed mice (E-H) were collected one week after the last booster injection and challenged with 20μg/ml tCRAMP for 24 hours and stained for T cell activation markers. Gating is as depicted in S3 Fig. Bar over graph columns indicate statistical significance; P<0.05. Mean of 3 separate experiments with at least 2 mice pooled per group in 3–6 replicates per experiment.

The immunologic changes in the T cell populations in immunized compared to control mice in response to 24-hour recall challenge with tCRAMP were then tested. Compared to controls, immunization with the Cramp 100μg dose significantly increased both CD4+ and CD8+ EM T cells (Fig 8A and 8C) suggesting exacerbated immune activation. In contrast to the changes seen in the 100μg dose, only CD8+ CM T cells were significantly increased in the 20μg immunization dose (Fig 8H), suggesting selectivity in the response.

### Dendritic cell profile of immunized mice

**Fig 9:**
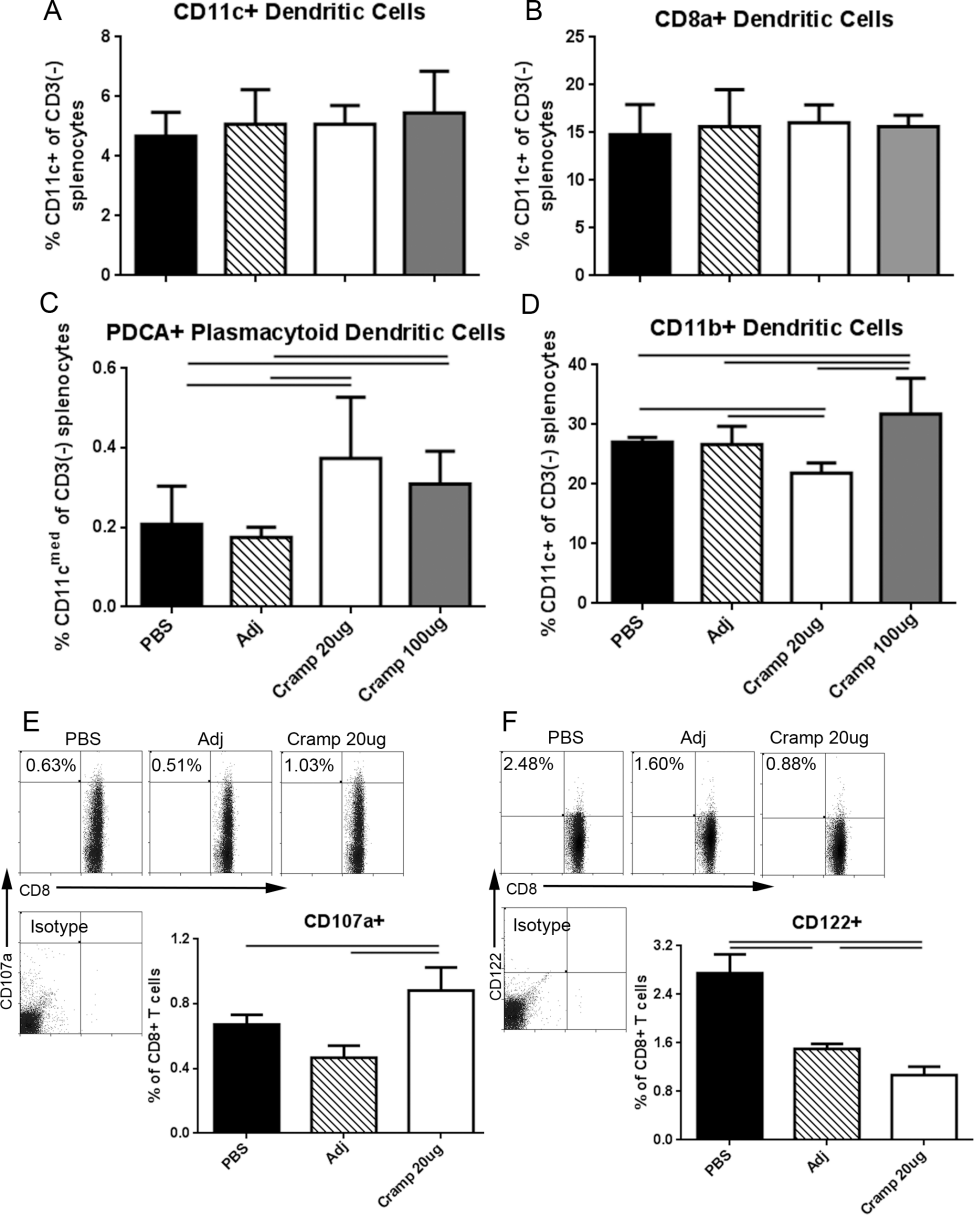
DC subtypes, CD8 cytotoxic degranulation, and CD8+CD122+ T_reg_ cells in splenocytes of immunized mice. Splenocytes from the different experimental groups were treated with 20μg/ml tCRAMP for 24 hours and stained for markers of DC subtypes (A-D). Gating is as depicted in S5 Fig. Bars over graph columns indicate statistical significance; P<0.05. Mean of 3–5 separate experiments with at least 2 mice pooled per group in 3–4 replicates per experiment. Splenocytes from mice immunized with 20μg Cramp, adjuvant, or PBS were treated with 20μg/ml tCRAMP for 4 hours in the presence of 2μg/ml fluorescently labeled CD107a monoclonal antibody. Cells were also stained for CD8b and CD122, and analyzed for CD107a+ degranulation (E) or CD8+CD122+ T_reg_ cells (F). Analysis excluded CD49b+ cells. Isotype staining was used as control. Results of the analysis were plotted on bar graphs. Bars over graph columns indicate statistical significance; P<0.05.

We next examined the dendritic cell (DC) population in the spleens of immunized mice euthanized 1 week after the 2^nd^ booster. Splenocytes from the various groups were challenged with tCRAMP for 24 hours. Gating scheme for DCs is shown in S5 Fig. No change was observed in the total CD11c+ DC population or in the CD8a+ conventional (c)DC subpopulation (Fig 9A and 9B, respectively). The CD11c^med/low^ plasmacytoid (p)DC population was significantly higher in both doses of Cramp immunized groups compared to controls (Fig 9C) suggesting this cell population unlikely to be involved in the differential outcome of the two doses used. Analysis of the CD11c+CD11b+ cDC population, on the other hand, revealed a significant reduction in the 20μg dosed group with a contrasting and significant increase in CD11b+ cDCs in the 100μg dosed group (Fig 9D). Thus, the increased CD11b+ cDC population in the 100μg dosed group accompanied by the increased EM T cells further supports an exacerbated immune activation. On the other hand, the reduction in CD11b+ cDCs in the 20μg dosed group needed further investigation.

### Increased CD8+ T cell degranulation in 20μg-dosed mice

To determine the potential mechanism of the reduction in CD11b+ cDC in the 20μg dosed group, we performed a 4h degranulation study utilizing CD107a as a marker of cytotoxic T cell degranulation. Stimulation of splenocytes with tCRAMP revealed a significant increase in degranulating CD8+ T cells from the 20μg dosed group compared to controls (Fig 9E), a change reciprocating the CD11b+ cDC reduction in the 20μg dosed mice. There was a concomitant reduction in CD8+CD122+ T_reg_ cells in the 20μg immunized mice (Fig 9F).

## Discussion

Self-reactive T cells are purged during maturation of the immune system, but a small number escapes into the periphery. In the context of atherosclerosis, prior reports provided evidence of LDL-reactive CD4+ and CD8+ T cells [2] [3]. Heat-shock proteins (HSP) have also been demonstrated to provoke T cell responses [4]. However, the diversity of the T cell receptor repertoire, which by its nature enables the recognition of a large number of antigens, suggests that other potential self-antigens may also play a role in atherosclerosis. Atherosclerosis is a multi-factorial disease [4], with various immune-inflammatory pathways and it is likely that only incremental alteration of the disease process can be achieved from immune intervention with a single self-antigen. Identification of other potential self-antigens is therefore important.

Self-antigens presented by MHC-I are important in the generation of normal immune homeostasis. The CD8+ T cell memory repertoire is generated and maintained in the context of self-reactivity, which is a determinant of normal immune functions [7,8,29]. Altered immune reactivity to self-antigens in diseases may occur in the context of the MHC-I/self-peptide-CD8+ T cell axis [10,11]. LL-37, the cationic peptide fragment of hCAP-18 [14], is a self-antigen [15] potentially involved in atherosclerosis. LL-37 is a key component of NETs [20] which are involved in athero-thrombotic events [30]. LL-37 has been detected in atherosclerotic plaques [17,18] and its presence in plasma is reported to be modulated during myocardial infarction [31]. LL-37 is a T cell auto-antigen in psoriatic patients [15] and there is increased prevalence of psoriasis in CAD patients [13], with increased risk for myocardial infarction [16]. Thus, it is possible that hCAP-18 fragments may be a source of auto-antigens in atherosclerosis. The mouse homolog of hCAP-18, called CRAMP, has been described as a pro-atherosclerotic molecule [32,33]. Genetically altered mice lacking CRAMP have reduced atherosclerotic burden [34].

We tested ApoE(-/-) mouse immune self-reactivity to CRAMP by first determining the MHC-I binding potential using in silico analysis (www.syfpeithi.de). After segments of the CRAMP protein with high binding prediction scores for H2-D^b^ and H2-K^b^ were identified, the regions with low binding prediction were excluded from the synthesized truncated CRAMP. This was done in order to test only regions of the CRAMP protein with the potential to stimulate CRAMP-reactive CD8+ T cells in the ApoE(-/-) mice through H2-D^b^ and H2-K^b^. Using splenocytes from naïve ApoE(-/-) mice, we observed CD8+ T cell reactivity to tCRAMP stimulation evidenced by the increase of cells with memory phenotype, and further increased when the mice were fed a high fat diet. Thus, we were able to provoke memory-like responses from CRAMP-reactive CD8+ T cells using the truncated CRAMP peptide that included high-scoring predictions for MHC-I binding regions. Importantly, these CRAMP-reactive memory-like CD8+ T cells were also found in atherosclerotic plaques in the aorta. The rapid response of these memory-like CD8+ T cells is consistent with the known properties of some CD8+ memory T cell subpopulations with rapid recall responses [35]. The results are in agreement with our recent report on the presence of self-reactive CD8+ T cells with memory phenotype specific for another self-peptide antigen derived from apolipoprotein B-100 [36].

We used immunization as a method to modify the immune response of ApoE(-/-) mice to the self-antigen. The truncated CRAMP peptide was useful in determining steady-state immune reactivity in the MHC-I/self-peptide-CD8+ T cell axis, but would not be appropriate to use as a self-antigen for immunization. The generation of tCRAMP peptide for in vitro challenge involved excluding fragments with low MHC-I binding potential and linking the remainder of the peptide sequence. These linked peptides have the potential to be recognized as foreign by the mouse immune system since it does not conform to the native protein. The objective was to alter the immune response to the self-protein, therefore we needed to keep the native protein sequence intact for the immunization studies. Since the first 37 amino-acids of the CRAMP protein did not contain high H2-D^b^ or H2-K^b^ binding sites, it was reasonable to exclude the segment during peptide synthesis. The peptide Cramp was then generated at a high purity with no foreign peptide sequences and used for immunization. The choice of Adjuphos with MPLA as adjuvant is based on the previously reported efficacy of this adjuvant formulation to preferentially increase CD8+ T cell responses in experimental conditions [37]. We chose 2 different dosing regiments to address the potential complexity of the effect of antigen dose in auto-immune responses [9,22–24].

Our results reveal an interesting yet contrasting effect of the 100μg immunization dose compared to the 20μg dose. Immunizing with the 100μg dose of Cramp triggered exaggerated immune activation indicated by increased CD4+ EM and CD8+ EM T cells, along with increased CD11b+ cDC subpopulation. This immunological change was associated with increased atherosclerotic plaque burden, evidenced by increased aortic sinus plaque size and increased neutrophil infiltration. The result of the 100μg immunization dose is consistent with the reported reactivity to LL-37 by both CD4+ T cells and CD8+ T cells from psoriasis patients [15]. On the other hand, immunizing with the 20μg dose revealed an intriguing effect on atherosclerosis with a significant reduction in aortic atherosclerosis burden.

The differential effect of the 2 doses of antigens used in the immunization studies underscores the complexity of self-antigenic responses. Low or moderate antigen exposure generates tolerance by high specificity reactive T cells. High antigenic exposure results in rapid deletion [24]. However, this occurs in the context of the inflammatory milieu and may not result in complete deletion of reactive cells if the inflammatory status is heightened. High antigen exposure in this situation can result in the activation of lower specificity T cells and thus feed-forward increased inflammatory signaling [9]. It has been reported that the amount of self-antigen determines the effector function of T cells [23]. Previous studies on the effect of antigen amount during immune activation used two different transgenic mouse lines that expressed truncated chicken ovalbumin (OVA) as a self-antigen in intestinal enterocytes at either low or high levels [8,9]. Although both mouse lines appeared normal at steady-state, infection with OVA-encoding vesicular stomatitis virus resulted in severe pathology after transfer of OT-I cells (OVA-specific CD8+ T cells) in the high OVA-expressing mouse line leading to the destruction of intestinal epithelium. The low OVA-expressing mouse line on the other hand showed only a mild, transient response and recovered rapidly [9]. Thus, self-reactive, high-specificity CD8+ T cells that are generated in response to low antigen amount in the context of an inflammatory response are kept under control. On the other hand, self-reactive, low-specificity CD8+ T cells that are generated in response to high antigen amount are retained to actively participate during immune challenges [8]. A similar outcome was reported in a Type 1 diabetes model where two transgenic mouse lines expressed different levels of a LCMV protein as self-antigen in beta cells of the pancreas. The mouse line that expressed high antigen amounts resulted in worse disease [22]. Although the lower antigen amount in the cited reports only showed marginal effect on the diseases studied, the lower dose in our study imparted a protective effect. It is possible that that the difference in the kinetics of the disease model studied affects the immune profiles. In support of this, it has been suggested that regulatory T cell response may not be effective in long-term chronic conditions [23]. Both intestinal epithelial destruction and onset of Type 1 diabetes occurred rapidly over 2–3 weeks. Atherosclerosis on the other hand, extends over a much longer period of time. In this context, a similar differential effect of dose on atherosclerosis was reported for a peptide derived from apolipoprotein B-100 called P6 [38,39]. The peptide was previously shown to reduce atherosclerosis in mice, using dose ranges similar to our study [38]. A second report by the same authors subsequently investigated the immunogenicity of the peptide in steady-state and performed immunization with a higher dose resulting in exacerbation of atherosclerosis [39]. Our results are in agreement with their reports.

Differential outcomes with different amounts of antigen is attributed in part to peripheral tolerance mechanisms, specifically CD4+ regulatory T cells. A report using transgenic mice expressing two different amounts of influenza HA as a self-protein under control of the CD11c promoter showed that low expression resulted in the generation of CD4+ T_reg_ cells whereas high expression was associated with stringent T cell negative selection resulting in poor T_reg_ development [23]. We therefore assessed whether the low-dosed mice in our study had any increase in CD4+ T_regs_. However, CD4+CD25+FoxP3+ T_reg_ cells or CD8+CD122+ T_reg_ cells showed a lack of selectivity for the 20μg dosed-group, suggesting that they are not involved in the protective effect on atherosclerosis.

While the exact mechanism underlying the protective effect of the lower dose in our study remains to be completely elucidated, our results show increased CD8+ CM T cell activation and degranulation [40] and a significant reduction in CD11b+ cDC suggesting the involvement of these cell populations. We showed that the CD11c+CD11b+ cDCs from high fat diet fed mice exhibit a pro-inflammatory cytokine profile with a significantly increased production of both IFN-≅γ and TNF-α after stimulation with CRAMP. CD11b+ cells are a major subtype of cDCs and are the most abundant population of DCs in atherosclerotic plaques [27,28] but their role in atherosclerosis is not yet clearly defined. However, they have been shown to rapidly increase during atherogenesis and are found in both mouse and human atherosclerotic plaques. CD11b plays a role in leukocyte adhesion, migration, activation and phagocytosis. CD11b deficiency in DCs leads to impairment of CD4+ T cell activation [41].

CD8+ T cell mediated killing of DCs is a potential mechanism of regulating immune responses to control against heightened inflammation [42]. Our results show increased CD8+ T cell degranulation as detected by CD107a staining concomitant with reduced CD11b+CD11c+ DCs, suggesting a role for this pathway in the favorable effect of the 20μg dose. The CD8+ T cell-DC interaction has been described as a potential regulatory loop for immune regulation [43]. The significant increase in CD11c+CD11b+ cDCs in the 100μg dosed group in our study suggests a potential pathogenic role for subtypes of these cDCs and provides additional evidence of the exaggerated immune activation following high dose Cramp immunization.

The role of antigen-presenting DCs in atherosclerosis is currently subject to increasing interest [44]. LL-37 activates pDCs through DNA/LL-37 complex formation [20], and pDCs are involved in atherosclerosis [32,45–49]. Our results confirm the role of CRAMP in the induction of pDCs seen in both immunization dose groups but the similar degree of change in pDCs in both dose groups in our study suggested that the differential effects on atherosclerosis are unlikely to be pDC dependent.

CD8a+ DCs are specialized for cross-presentation of necrotic tissue-derived antigens to activate CD8+ T cells, are Batf3-dependent, and are important for peripheral immune homeostasis. In agreement with a previous report showing that loss of CD8a+ DCs did not affect atherosclerosis in mice [50], immunization did not alter CD8a+ DCs in our study. It is likely that the antigen source, context of antigen exposure, and specific disease milieu are important factors in determining the role of DC subtypes in atherosclerosis.

The lack of difference in anti-Cramp IgG antibodies generated by the immunized mice in both the 20μg and in the 100μg dose suggests that antibodies do not have a direct role. Interestingly, although LL-37 was reported as a T cell antigen in psoriasis patients, no evidence of antibody response was provided [15]. There were also no differences in serum cholesterol levels among the groups suggesting that the effects on atherosclerosis were mediated mainly through immune-inflammatory responses.

A limitation of the study is the lack of mechanistic insight on how self-reactivity becomes a determinant in a chronic disease such as atherosclerosis. Previous reports suggest that the magnitude of inflammatory signaling skews the immune system that can result in this outcome [9,24]. How this specifically relates to atherosclerosis needs to be further elucidated. There is also the relative difference in the actual disease in humans compared to the mouse disease model. The development of atherosclerosis in ApoE(-/-) mice occurs over weeks compared to several decades in humans. However, the presence of immuno-inflammatory responses to atherosclerosis are comparable between the human and ApoE(-/-) model.

CAD is a condition that is manifested by atherosclerotic plaque formation and burden. Auto-immune, inflammatory diseases such as psoriasis are linked to increased severity of CAD. The hCAP-18 fragment LL-37 is a known autoantigen in psoriasis patients. Relevance of auto-reactive self-antigens to atherosclerotic disease was tested using the mouse homolog CRAMP in ApoE(-/-) mice. Our results show that the memory profile of CRAMP-reactive T cells are changed by high fat diet feeding, and is affected by antigen dose used for immunization with differential effects on atherosclerosis. It would now be interesting to investigate the relationship between immune reactivity to LL-37 or other fragments of hCAP-18, and atherosclerosis in psoriasis patients. Our report will aid in identifying novel pathways of immune signaling in response to self-antigens in atherosclerosis.

## Supporting information

S1 Fig The mouse CRAMP sequence was assessed for predicted MHC-I binding. A truncated peptide (tCRAMP) was generated excluding segments with low MHC-I binding prediction for use in cell stimulation. The peptide used for immunization (Cramp) was synthesized excluding the first 37 amino acids because of low MHC-I binding prediction, but was identical with the rest of the CRAMP sequence in order to maintain properties of self-peptides.
(TIF)

S2 Fig The peptide derived from mouse serum Albumin contained segments with moderate MHC-I binding prediction. (TIF)

S3 Fig Gating scheme for analysis of T cell populations in splenocytes. The gating scheme depicted (A) is used for all T cell analysis throughout the report. Prior to the size-gating with FSC vs SSC, cell doublets and non-viable cells were selected out as dump gates. Size-gated cells were then plotted on CD4+ vs CD8b+ and used for analysis for CD44 and CD62L staining. CD4+ T cells were further plotted on CD25+ vs FoxP3, which is GFP+. Isotypes were used as references for the cell stains. Splenocytes from WT mice were used as reference for FoxP3 expression. Representative plot of intra-cellular IFN-γ staining in T cells as gated from CD8+ or CD4+ cells (B). Representative histogram of CFSE labeled cells as a measure of proliferating cells gated for CD8+ or CD4+ T cells (C).
(TIF)

S4 Fig Stimulation of splenocytes from mice fed high fat diet. Splenocytes from naive ApoE(-/-) mice fed a high fat diet for 6 weeks were stimulated for 24 hours with either mouse serum Albumin peptide or tCRAMP (20mg/ml each). There was increased Effector Memory (EM) and Central Memory (CM) CD8+T cells (A and B, respectively) after tCRAMP stimulation but no effect by Albumin peptide stimulation. EM and CM CD4+ T cells (C and D, respectively) were significantly reduced after tCRAMP stimulation but Albumin peptide had no effect. Analysis of cell stains was based on the gating scheme depicted in S3 Fig. Bars over graphed columns indicate statistical significance (P<0.05; N = 4 each).
(TIF)

S5 Fig Gating scheme for dendritic cell (DC) analysis in splenocytes. The gating scheme depicted is used for all DC analysis throughout the report. Prior to the size-gating with FSC vs SSC, cell doublets, non-viable cells, and CD3e+ cells were selected out as dump gates. PDCA+ pDCs were determined based on size gated cells plotted as CD11c med/low (top right panel). CD8a+ conventional (c) DCs (middle panels) and CD11b+ cDCs (middle and bottom left panels) were size-gated and selected for CD11c+ staining. Isotype stained cells were used as reference.
(TIF)

S6 Fig Negative controls for immuno-histochemical staining. Staining control for macrophages (A), neutrophil (B) and CD3 (C) as validation of specific stains in Fig 6.
(TIF)

## References

[1] Nilsson J, Hansson GK. Autoimmunity in atherosclerosis: a protective response losing control? J Intern Med. 2008;263(5): 464–478. doi: 10.1111/j.1365-2796.2008.01945.x 18410590 10.1111/j.1365-2796.2008.01945.x

[2] Zhou X, Nicoletti A, Elhage R, Hansson GK. Transfer of CD4(+) T cells aggravates atherosclerosis in immunodeficient apolipoprotein E knockout mice. Circulation. 2000;102(24): 2919–2922. 11113040 10.1161/01.cir.102.24.2919

[3] Stemme S, Faber B, Holm J, Wiklund O, Witztum JL, Hansson GK. T lymphocytes from human atherosclerotic plaques recognize oxidized low density lipoprotein. Proc Natl Acad Sci U S A. 1995;92(9): 3893–3897. 7732003 10.1073/pnas.92.9.3893 PMC42068

[4] Almanzar G, Ollinger R, Leuenberger J, Onestingel E, Rantner B, Zehm S, et al Autoreactive HSP60 epitope-specific T-cells in early human atherosclerotic lesions. J Autoimmun. 2012;39: 441–450. doi: 10.1016/j.jaut.2012.07.006 22901435 10.1016/j.jaut.2012.07.006 PMC3516706

[5] Nilsson J, Bjorkbacka H, Fredrikson GN. Apolipoprotein B100 autoimmunity and atherosclerosis—disease mechanisms and therapeutic potential. Curr Opin Lipidol. 2012;23(5): 422–428. doi: 10.1097/MOL.0b013e328356ec7c 22814703 10.1097/MOL.0b013e328356ec7c

[6] Bourdetsky D, Schmelzer CE, Admon A. The nature and extent of contributions by defective ribosome products to the HLA peptidome. Proc Natl Acad Sci U S A. 2014;111(16): E1591–E1599. doi: 10.1073/pnas.1321902111 24715725 10.1073/pnas.1321902111 PMC4000780

[7] Dhanji S, Chow MT, Teh HS. Self-antigen maintains the innate antibacterial function of self-specific CD8 T cells in vivo. J Immunol. 2006;177: 138–146. 16785508 10.4049/jimmunol.177.1.138

[8] Turner MJ, Jellison ER, Lingenheld EG, Puddington L, Lefrancois L. Avidity maturation of memory CD8 T cells is limited by self-antigen expression. J Exp Med. 2008;205(8): 1859–1868. doi: 10.1084/jem.20072390 18625745 10.1084/jem.20072390 PMC2525599

[9] Vezys V, Olson S, Lefrancois L. Expression of intestine-specific antigen reveals novel pathways of CD8 T cell tolerance induction. Immunity. 2000;12(5): 505–514. 10843383 10.1016/s1074-7613(00)80202-2

[10] Bassani-Sternberg M, Barnea E, Beer I, Avivi I, Katz T, Admon A. Soluble plasma HLA peptidome as a potential source for cancer biomarkers. Proc Natl Acad Sci U S A. 2010;107(44): 18769–18776. doi: 10.1073/pnas.1008501107 20974924 10.1073/pnas.1008501107 PMC2973870

[11] Hickman HD, Yewdell JW. Mining the plasma immunopeptidome for cancer peptides as biomarkers and beyond. Proc Natl Acad Sci U S A. 2010;107(44): 18747–18748. doi: 10.1073/pnas.1013851107 20974971 10.1073/pnas.1013851107 PMC2973875

[12] Santilli S, Kast DR, Grozdev I, Cao L, Feig RL, Golden JB, et al Visualization of atherosclerosis as detected by coronary artery calcium and carotid intima-media thickness reveals significant atherosclerosis in a cross-sectional study of psoriasis patients in a tertiary care center. J Transl Med. 2016;14(1): 217–0947. doi: 10.1186/s12967-016-0947-0 27448600 10.1186/s12967-016-0947-0 PMC4957305

[13] Picard D, Benichou J, Sin C, Abasq C, Houivet E, Koning R, et al Increased prevalence of psoriasis in patients with coronary artery disease: results from a case-control study. Br J Dermatol. 2014;171(3): 580–587. doi: 10.1111/bjd.13155 24904002 10.1111/bjd.13155

[14] Sorensen OE, Follin P, Johnsen AH, Calafat J, Tjabringa GS, Hiemstra PS, et al Human cathelicidin, hCAP-18, is processed to the antimicrobial peptide LL-37 by extracellular cleavage with proteinase 3. Blood. 2001;97(12): 3951–3959. 11389039 10.1182/blood.v97.12.3951

[15] Lande R, Botti E, Jandus C, Dojcinovic D, Fanelli G, Conrad C, et al The antimicrobial peptide LL37 is a T-cell autoantigen in psoriasis. Nat Commun. 2014;5: 5621 doi: 10.1038/ncomms6621 25470744 10.1038/ncomms6621

[16] Gelfand JM, Neimann AL, Shin DB, Wang X, Margolis DJ, Troxel AB. Risk of myocardial infarction in patients with psoriasis. JAMA. 2006;296(14): 1735–1741. doi: 10.1001/jama.296.14.1735 17032986 10.1001/jama.296.14.1735

[17] Edfeldt K, Agerberth B, Rottenberg ME, Gudmundsson GH, Wang XB, Mandal K, et al Involvement of the antimicrobial peptide LL-37 in human atherosclerosis. Arterioscler Thromb Vasc Biol. 2006;26(7): 1551–1557. doi: 10.1161/01.ATV.0000223901.08459.57 16645154 10.1161/01.ATV.0000223901.08459.57

[18] Ciornei CD, Tapper H, Bjartell A, Sternby NH, Bodelsson M. Human antimicrobial peptide LL-37 is present in atherosclerotic plaques and induces death of vascular smooth muscle cells: a laboratory study. BMC Cardiovasc Disord. 2006;20;6: 49 doi: 10.1186/1471-2261-6-20 17181861 10.1186/1471-2261-6-49 PMC1764755

[19] Ahlehoff O, Gislason GH, Lindhardsen J, Olesen JB, Charlot M, Skov L, et al Prognosis following first-time myocardial infarction in patients with psoriasis: a Danish nationwide cohort study. J Intern Med. 2011;270(3): 237–244. doi: 10.1111/j.1365-2796.2011.02368.x 21362070 10.1111/j.1365-2796.2011.02368.x

[20] Lande R, Ganguly D, Facchinetti V, Frasca L, Conrad C, Gregorio J, et al Neutrophils activate plasmacytoid dendritic cells by releasing self-DNA-peptide complexes in systemic lupus erythematosus. Sci Transl Med. 2011;3(73): 73ra19 doi: 10.1126/scitranslmed.3001180 21389263 10.1126/scitranslmed.3001180 PMC3399524

[21] Kahlenberg JM, Kaplan MJ. Little peptide, big effects: the role of LL-37 in inflammation and autoimmune disease. J Immunol. 2013;191(10): 4895–4901. doi: 10.4049/jimmunol.1302005 24185823 10.4049/jimmunol.1302005 PMC3836506

[22] Martinic MM, Huber C, Coppieters K, Oldham JE, Gavin AL, von Herrath MG. Expression level of a pancreatic neo-antigen in beta cells determines degree of diabetes pathogenesis. J Autoimmun. 2010;35(4): 404–413. doi: 10.1016/j.jaut.2010.08.006 20932718 10.1016/j.jaut.2010.08.006 PMC2963714

[23] Swee LK, Nusser A, Curti M, Kreuzaler M, Rolink H, Terracciano L, et al The amount of self-antigen determines the effector function of murine T cells escaping negative selection. Eur J Immunol. 2014;44(5): 1299–1312. doi: 10.1002/eji.201343840 24497074 10.1002/eji.201343840

[24] Kurts C, Sutherland RM, Davey G, Li M, Lew AM, Blanas E, et al CD8 T cell ignorance or tolerance to islet antigens depends on antigen dose. Proc Natl Acad Sci U S A. 1999;96(22): 12703–12707. 10535986 10.1073/pnas.96.22.12703 PMC23058

[25] Zhou J, Dimayuga PC, Zhao X, Yano J, Lio WM, Trinidad P, et al CD8(+)CD25(+) T cells reduce atherosclerosis in apoE(-/-) mice. Biochem Biophys Res Commun. 2014;443(3): 864–870. doi: 10.1016/j.bbrc.2013.12.057 24342615 10.1016/j.bbrc.2013.12.057

[26] Chyu KY, Zhao X, Dimayuga PC, Zhou J, Li X, Yano J, et al CD8 T Cells Mediate the Athero-Protective Effect of Immunization with an ApoB-100 Peptide. PLoS One. 2012;7: e30780 doi: 10.1371/journal.pone.0030780 22347402 10.1371/journal.pone.0030780 PMC3276497

[27] Van B, I, Ammi R, Rombouts M, Cools N, Vercauteren SR, De RD, et al Fluorescent activated cell sorting: an effective approach to study dendritic cell subsets in human atherosclerotic plaques. J Immunol Methods. 2015;417: 76–85. doi: 10.1016/j.jim.2014.12.010 25527343 10.1016/j.jim.2014.12.010

[28] Rombouts M, Ammi R, Van B, I, Roth L, De Winter BY, Vercauteren SR, et al Linking CD11b (+) Dendritic Cells and Natural Killer T Cells to Plaque Inflammation in Atherosclerosis. Mediators Inflamm. 2016;2016: 6467375 doi: 10.1155/2016/6467375 27051078 10.1155/2016/6467375 PMC4804096

[29] Haluszczak C, Akue AD, Hamilton SE, Johnson LD, Pujanauski L, Teodorovic L, et al The antigen-specific CD8+ T cell repertoire in unimmunized mice includes memory phenotype cells bearing markers of homeostatic expansion. J Exp Med. 2009;206(2): 435–448. doi: 10.1084/jem.20081829 19188498 10.1084/jem.20081829 PMC2646575

[30] Mangold A, Alias S, Scherz T, Hofbauer T, Jakowitsch J, Panzenbock A, et al Coronary neutrophil extracellular trap burden and deoxyribonuclease activity in ST-elevation acute coronary syndrome are predictors of ST-segment resolution and infarct size. Circ Res. 2015;116(7): 1182–1192. doi: 10.1161/CIRCRESAHA.116.304944 25547404 10.1161/CIRCRESAHA.116.304944

[31] Zhao H, Yan H, Yamashita S, Li W, Liu C, Chen Y, et al Acute ST-segment elevation myocardial infarction is associated with decreased human antimicrobial peptide LL-37 and increased human neutrophil peptide-1 to 3 in plasma. J Atheroscler Thromb. 2012;19(4): 357–368. 22186100 10.5551/jat.10108

[32] Doring Y, Manthey HD, Drechsler M, Lievens D, Megens RT, Soehnlein O, et al Auto-antigenic protein-DNA complexes stimulate plasmacytoid dendritic cells to promote atherosclerosis. Circulation. 2012;125(13): 1673–1683. doi: 10.1161/CIRCULATIONAHA.111.046755 22388324 10.1161/CIRCULATIONAHA.111.046755

[33] Zhang Z, Meng P, Han Y, Shen C, Li B, Hakim MA, et al Mitochondrial DNA-LL-37 Complex Promotes Atherosclerosis by Escaping from Autophagic Recognition. Immunity. 2015;43(6): 1137–1147. doi: 10.1016/j.immuni.2015.10.018 26680206 10.1016/j.immuni.2015.10.018

[34] Doring Y, Drechsler M, Wantha S, Kemmerich K, Lievens D, Vijayan S, et al Lack of neutrophil-derived CRAMP reduces atherosclerosis in mice. Circ Res. 2012;110(8): 1052–1056. doi: 10.1161/CIRCRESAHA.112.265868 22394519 10.1161/CIRCRESAHA.112.265868

[35] DiSpirito JR, Shen H. Quick to remember, slow to forget: rapid recall responses of memory CD8+ T cells. Cell Res. 2010;20(1): 13–23. doi: 10.1038/cr.2009.140 20029390 10.1038/cr.2009.140

[36] Dimayuga PC, Zhao X, Yano J, Lio WM, Zhou J, Mihailovic PM, et al Identification of apoB-100 Peptide-specific CD8+ T cells in Atherosclerosis. J Am Heart Assoc. 2017 7 15;6(7). pii: e005318 doi: 10.1161/JAHA.116.005318 28711866 10.1161/JAHA.116.005318 PMC5586274

[37] MacLeod MK, McKee AS, David A, Wang J, Mason R, Kappler JW, et al Vaccine adjuvants aluminum and monophosphoryl lipid A provide distinct signals to generate protective cytotoxic memory CD8 T cells. Proc Natl Acad Sci U S A. 2011;108(19): 7914–7919. doi: 10.1073/pnas.1104588108 21518876 10.1073/pnas.1104588108 PMC3093483

[38] Tse K, Gonen A, Sidney J, Ouyang H, Witztum JL, Sette A, et al Atheroprotective Vaccination with MHC-II Restricted Peptides from ApoB-100. Front Immunol. 2013;4: 493 doi: 10.3389/fimmu.2013.00493 24416033 10.3389/fimmu.2013.00493 PMC3873602

[39] Shaw MK, Tse KY, Zhao X, Welch K, Eitzman DT, Thipparthi RR, et al T-Cells Specific for a Self-Peptide of ApoB-100 Exacerbate Aortic Atheroma in Murine Atherosclerosis. Front Immunol. 2017;8: 95 doi: 10.3389/fimmu.2017.00095 28280493 10.3389/fimmu.2017.00095 PMC5322236

[40] Betts MR, Brenchley JM, Price DA, De Rosa SC, Douek DC, Roederer M, et al Sensitive and viable identification of antigen-specific CD8+ T cells by a flow cytometric assay for degranulation. J Immunol Methods. 2003;281(1–2): 65–78. 14580882 10.1016/s0022-1759(03)00265-5

[41] Wu H, Rodgers JR, Perrard XY, Perrard JL, Prince JE, Abe Y, et al Deficiency of CD11b or CD11d results in reduced staphylococcal enterotoxin-induced T cell response and T cell phenotypic changes. J Immunol. 2004;173(1): 297–306. 15210787 10.4049/jimmunol.173.1.297

[42] Chen M, Felix K, Wang J. Critical role for perforin and Fas-dependent killing of dendritic cells in the control of inflammation. Blood. 2012;119(1): 127–136. doi: 10.1182/blood-2011-06-363994 22042696 10.1182/blood-2011-06-363994 PMC3251225

[43] Terrell CE, Jordan MB. Perforin deficiency impairs a critical immunoregulatory loop involving murine CD8(+) T cells and dendritic cells. Blood. 2013;121(26): 5184–5191. doi: 10.1182/blood-2013-04-495309 23660960 10.1182/blood-2013-04-495309 PMC3695362

[44] Paulson KE, Zhu SN, Chen M, Nurmohamed S, Jongstra-Bilen J, Cybulsky MI. Resident intimal dendritic cells accumulate lipid and contribute to the initiation of atherosclerosis. Circ Res. 2010;106(2): 383–390. doi: 10.1161/CIRCRESAHA.109.210781 19893012 10.1161/CIRCRESAHA.109.210781

[45] Van Vre EA, Hoymans VY, Bult H, Lenjou M, Van Bockstaele DR, Vrints CJ, et al Decreased number of circulating plasmacytoid dendritic cells in patients with atherosclerotic coronary artery disease. Coron Artery Dis. 2006;17(3): 243–248. 16728874 10.1097/00019501-200605000-00007

[46] Daissormont IT, Christ A, Temmerman L, Sampedro MS, Seijkens T, Manca M, et al Plasmacytoid dendritic cells protect against atherosclerosis by tuning T-cell proliferation and activity. Circ Res. 2011;109(12): 1387–1395. doi: 10.1161/CIRCRESAHA.111.256529 22021930 10.1161/CIRCRESAHA.111.256529 PMC3237962

[47] Yun TJ, Lee JS, Machmach K, Shim D, Choi J, Wi YJ, et al Indoleamine 2,3-Dioxygenase-Expressing Aortic Plasmacytoid Dendritic Cells Protect against Atherosclerosis by Induction of Regulatory T Cells. Cell Metab. 2016;23(5): 852–866. doi: 10.1016/j.cmet.2016.04.010 27166946 10.1016/j.cmet.2016.04.010

[48] Macritchie N, Grassia G, Sabir SR, Maddaluno M, Welsh P, Sattar N, et al Plasmacytoid dendritic cells play a key role in promoting atherosclerosis in apolipoprotein E-deficient mice. Arterioscler Thromb Vasc Biol. 2012;32(11): 2569–2579. doi: 10.1161/ATVBAHA.112.251314 22936340 10.1161/ATVBAHA.112.251314

[49] Sage AP, Murphy D, Maffia P, Masters LM, Sabir SR, Baker LL, et al MHC Class II-restricted antigen presentation by plasmacytoid dendritic cells drives proatherogenic T cell immunity. Circulation. 2014;130(16): 1363–1373. doi: 10.1161/CIRCULATIONAHA.114.011090 25223984 10.1161/CIRCULATIONAHA.114.011090 PMC4428652

[50] Legein B, Janssen EM, Theelen TL, Gijbels MJ, Walraven J, Klarquist JS, et al Ablation of CD8alpha(+) dendritic cell mediated cross-presentation does not impact atherosclerosis in hyperlipidemic mice. Sci Rep. 2015;5: 15414 doi: 10.1038/srep15414 26486587 10.1038/srep15414 PMC4614009

