# Supplementary figures and images for: The cathelicidin protein CRAMP is a potential atherosclerosis self-antigen in ApoE(-/-) mice
Source: PLoS One. 2017 Nov 1;12(11):e0187432. doi: 10.1371/journal.pone.0187432 (PMC5665601; doi:10.1371/journal.pone.0187432)

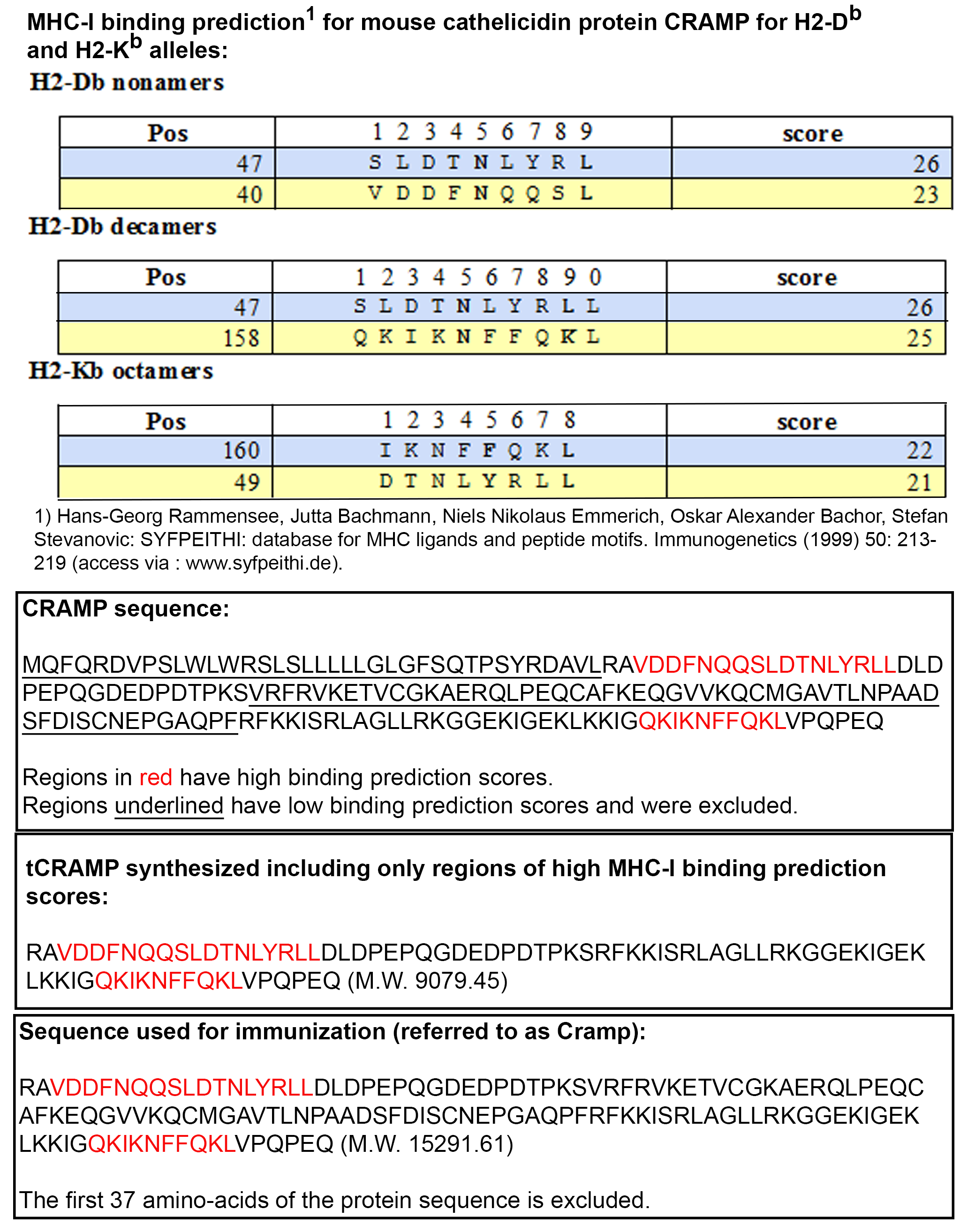

Supplement: S1 Fig — A truncated peptide (tCRAMP) was generated excluding segments with low MHC-I binding prediction for use in cell stimulation. The peptide used for immunization (Cramp) was synthesized excluding the first 37 amino acids because of low MHC-I binding prediction, but was identical with the rest of the CRAMP sequence in order to maintain properties of self-peptides. (TIF) [file pone.0187432.s001.tif]

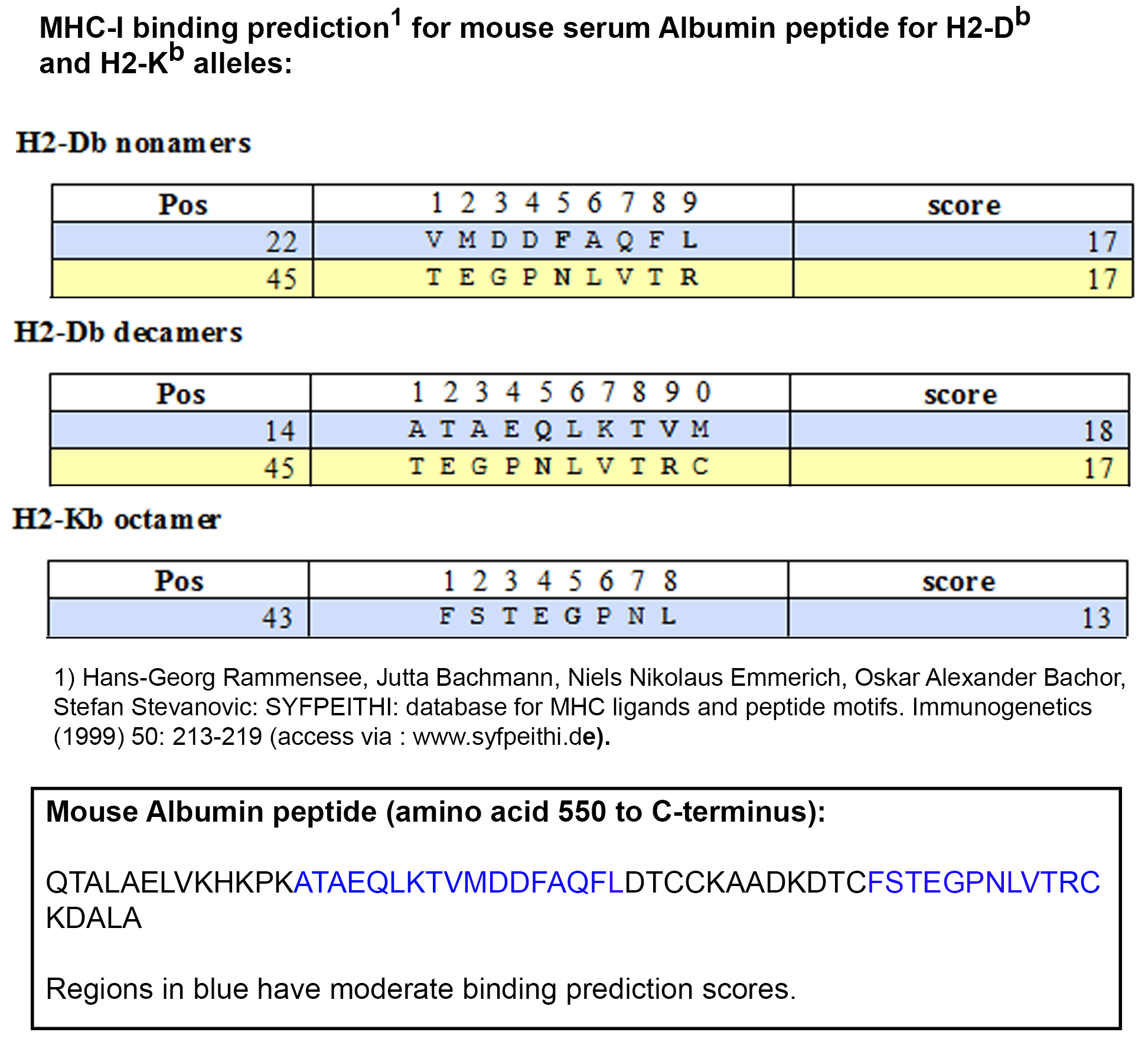

Supplement: S2 Fig — (TIF) [file pone.0187432.s002.tif]

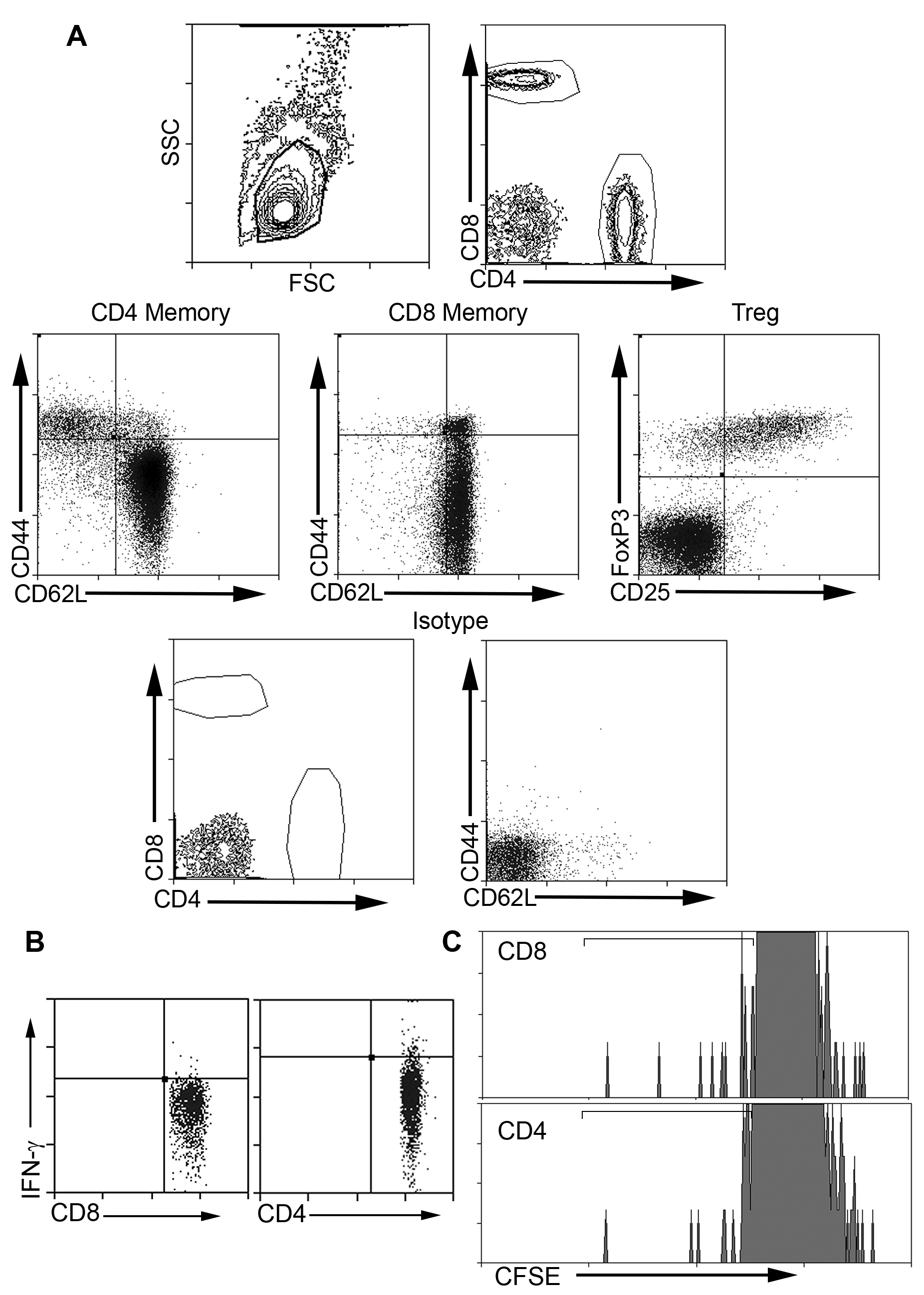

Supplement: S3 Fig — The gating scheme depicted (A) is used for all T cell analysis throughout the report. Prior to the size-gating with FSC vs SSC, cell doublets and non-viable cells were selected out as dump gates. Size-gated cells were then plotted on CD4+ vs CD8b+ and used for analysis for CD44 and CD62L staining. CD4+ T cells were further plotted on CD25+ vs FoxP3, which is GFP+. Isotypes were used as references for the cell stains. Splenocytes from WT mice were used as reference for FoxP3 expression. Representative plot of intra-cellular IFN-γ staining in T cells as gated from CD8+ or CD4+ cells (B). Representative histogram of CFSE labeled cells as a measure of proliferating cells gated for CD8+ or CD4+ T cells (C). (TIF) [file pone.0187432.s003.tif]

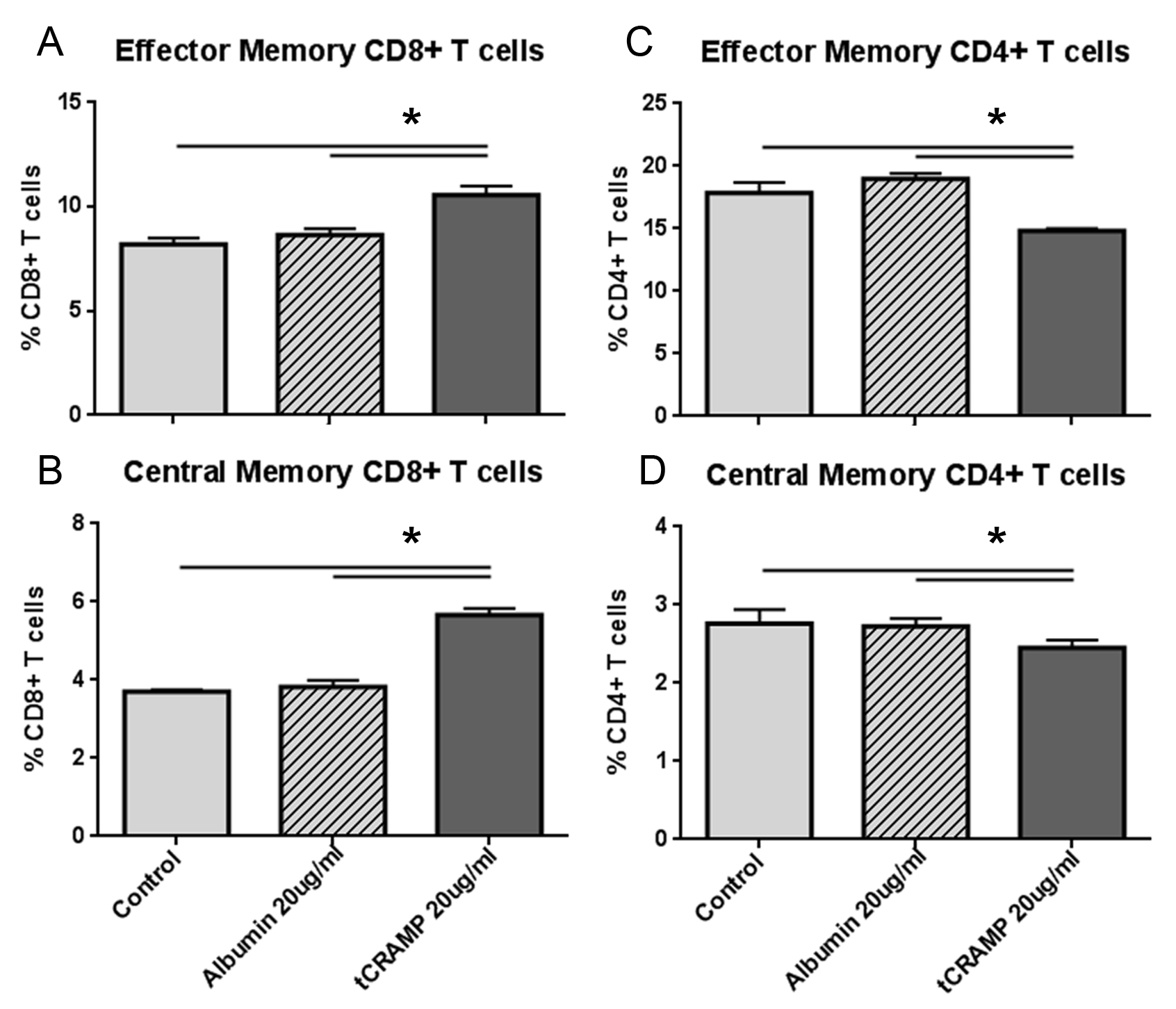

Supplement: S4 Fig — Splenocytes from naive ApoE(-/-) mice fed a high fat diet for 6 weeks were stimulated for 24 hours with either mouse serum Albumin peptide or tCRAMP (20mg/ml each). There was increased Effector Memory (EM) and Central Memory (CM) CD8+T cells (A and B, respectively) after tCRAMP stimulation but no effect by Albumin peptide stimulation. EM and CM CD4+ T cells (C and D, respectively) were significantly reduced after tCRAMP stimulation but Albumin peptide had no effect. Analysis of cell stains was based on the gating scheme depicted in S3 Fig. Bars over graphed columns indicate statistical significance (P<0.05; N = 4 each). (TIF) [file pone.0187432.s004.tif]

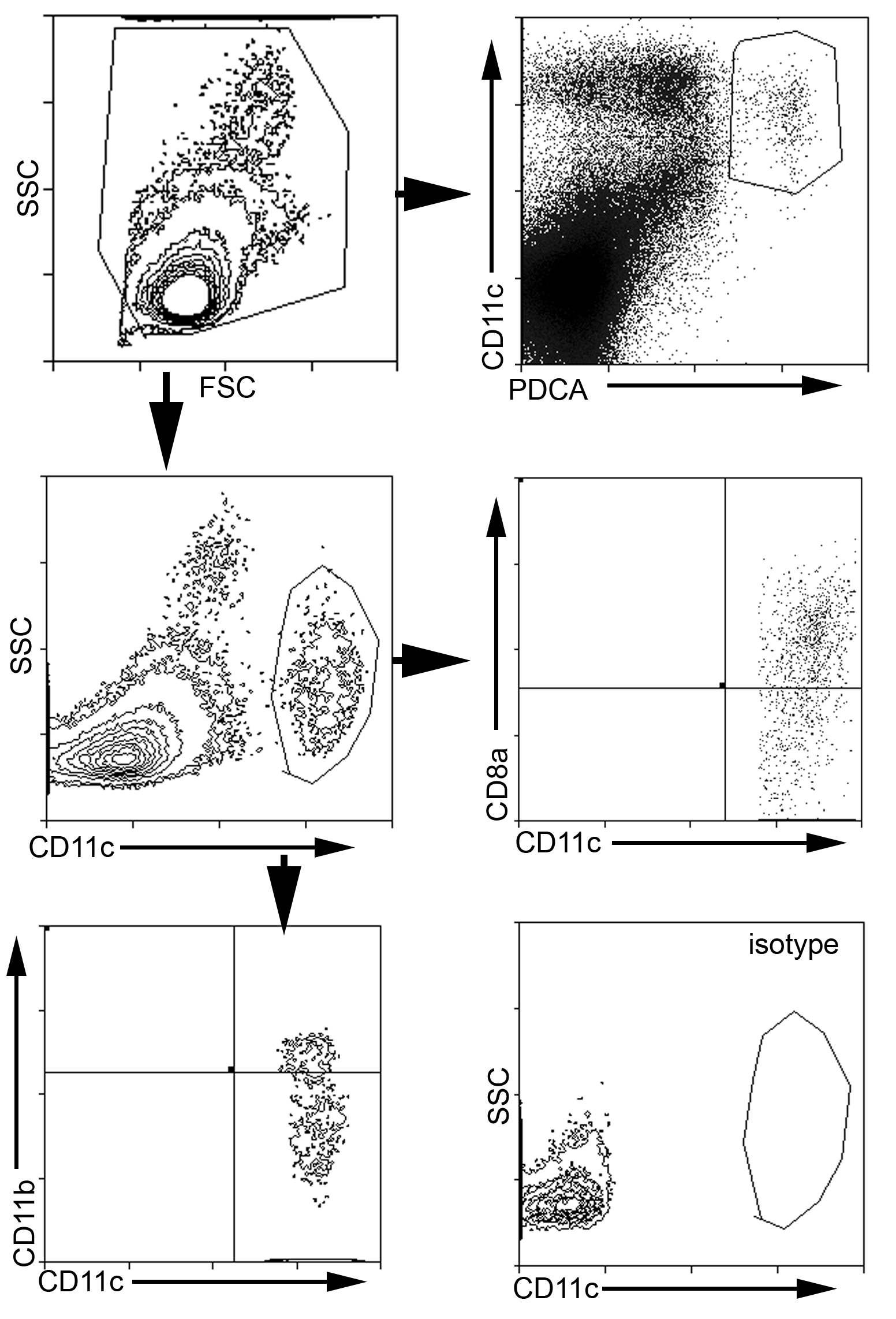

Supplement: S5 Fig — The gating scheme depicted is used for all DC analysis throughout the report. Prior to the size-gating with FSC vs SSC, cell doublets, non-viable cells, and CD3e+ cells were selected out as dump gates. PDCA+ pDCs were determined based on size gated cells plotted as CD11c med/low (top right panel). CD8a+ conventional (c) DCs (middle panels) and CD11b+ cDCs (middle and bottom left panels) were size-gated and selected for CD11c+ staining. Isotype stained cells were used as reference. (TIF) [file pone.0187432.s005.tif]

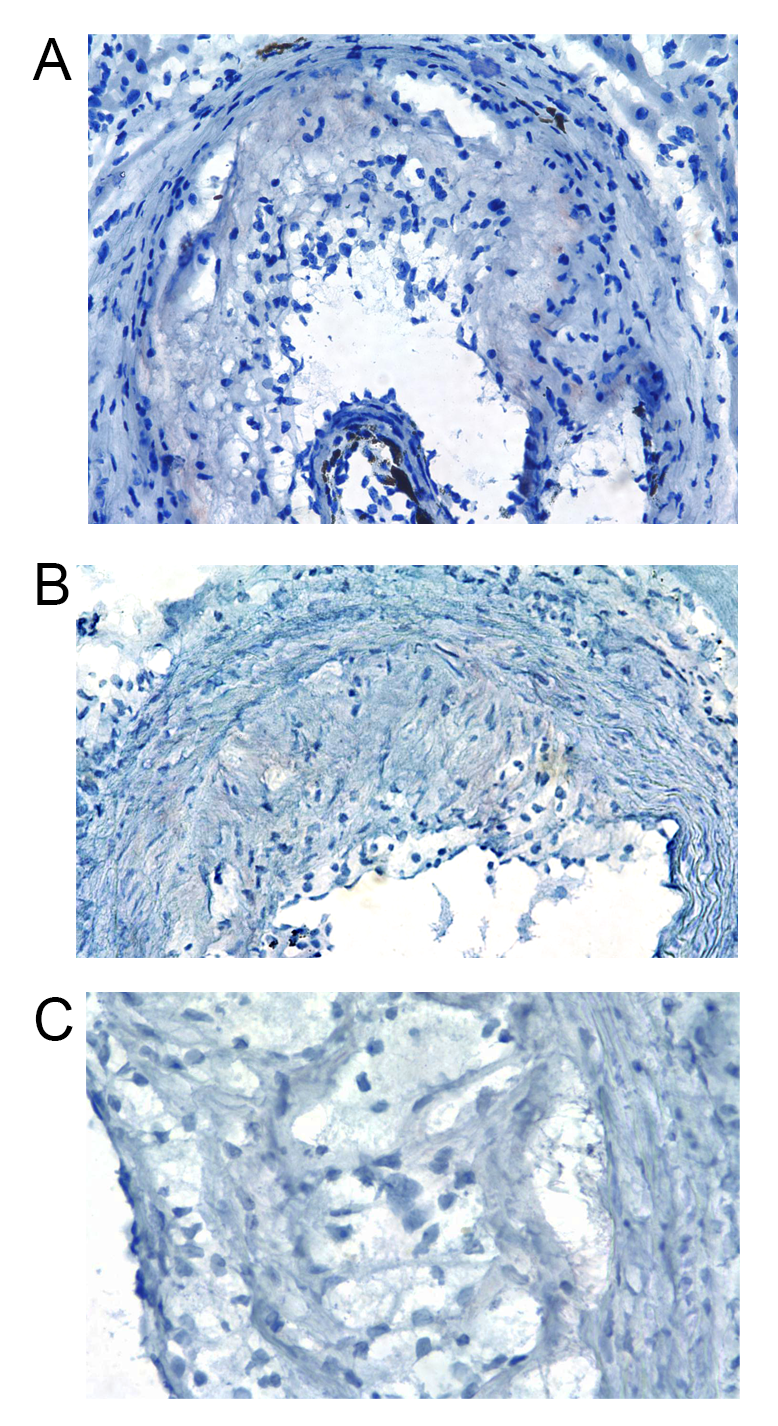

Supplement: S6 Fig — Staining control for macrophages (A), neutrophil (B) and CD3 (C) as validation of specific stains in Fig 6. (TIF) [file pone.0187432.s006.tif]
